# A comprehensive evaluation of SAM, the SAM R-package and a simple modification to improve its performance

**DOI:** 10.1186/1471-2105-8-230

**Published:** 2007-06-29

**Authors:** Shunpu Zhang

**Affiliations:** 1 Department of Statistics, University of Nebraska Lincoln, Lincoln, NE 68583-0963, USA

## Abstract

**Background:**

The Significance Analysis of Microarrays (SAM) is a popular method for detecting significantly expressed genes and controlling the false discovery rate (FDR). Recently, it has been reported in the literature that the FDR is not well controlled by SAM. Due to the vast application of SAM in microarray data analysis, it is of great importance to have an extensive evaluation of SAM and its associated R-package (sam2.20).

**Results:**

Our study has identified several discrepancies between SAM and sam2.20. One major difference is that SAM and sam2.20 use different methods for estimating FDR. Such discrepancies may cause confusion among the researchers who are using SAM or are developing the SAM-like methods. We have also shown that SAM provides no meaningful estimates of FDR and this problem has been corrected in sam2.20 by using a different formula for estimating FDR. However, we have found that, even with the improvement sam2.20 has made over SAM, sam2.20 may still produce erroneous and even conflicting results under certain situations. Using an example, we show that the problem of sam2.20 is caused by its use of asymmetric cutoffs which are due to the large variability of null scores at both ends of the order statistics. An obvious approach without the complication of the order statistics is the conventional symmetric cutoff method. For this reason, we have carried out extensive simulations to compare the performance of sam2.20 and the symmetric cutoff method. Finally, a simple modification is proposed to improve the FDR estimation of sam2.20 and the symmetric cutoff method.

**Conclusion:**

Our study shows that the most serious drawback of SAM is its poor estimation of FDR. Although this drawback has been corrected in sam2.20, the control of FDR by sam2.20 is still not satisfactory. The comparison between sam2.20 and the symmetric cutoff method reveals that the relative performance of sam2.20 to the symmetric cutff method depends on the ratio of induced to repressed genes in a microarray data, and is also affected by the ratio of DE to EE genes and the distributions of induced and repressed genes. Numerical simulations show that the symmetric cutoff method has the biggest advantage over sam2.20 when there are equal number of induced and repressed genes (i.e., the ratio of induced to repressed genes is 1). As the ratio of induced to repressed genes moves away from 1, the advantage of the symmetric cutoff method to sam2.20 is gradually diminishing until eventually sam2.20 becomes significantly better than the symmetric cutoff method when the differentially expressed (DE) genes are either all induced or all repressed genes. Simulation results also show that our proposed simple modification provides improved control of FDR for both sam2.20 and the symmetric cutoff method.

## 1 Background

Determination of significantly differentially expressed genes from replicated microarray data using nonparametric approaches has attracted much attention in recent years. A comprehensive review of earlier methods for processing and analyzing gene expression data generated using microarrays can be found in [1]. Generally speaking, the statistical methods used to detect differentially expressed genes can be classified into two categories: the parametric methods and the nonparametric methods. The most commonly used parametric method is the two sample *t*-test and its variations [2]. Other parametric approaches include the analysis of variance approach [3], a regression approach [4] and the empirical Bayes methods [5-7], among others. A semiparametric hierarchical mixture method for detecting differentially expressed genes was considered in [8].

Recently, there is growing interest in developing nonparametric methods in microarray data analysis due to the availability of replicated microarray data as a result of the reduced cost in doing microarray experiments. One of the most often used nonparametric methods for analyzing microarrays is SAM [9]. Other popular nonparametric methods include the nonparametric empirical Bayes method [10,11], and the mixture model method (MMM) [12], among others. In general, the nonparametric methods can be further classified into two categories: 1) methods such as SAM which provide direct control of FDR, and 2) methods such as MMM which provide control on the family wise error rate (FWER).

The focus of this paper is on SAM and its R-package (sam2.20) developed at Stanford University Labs [13]. As we will show later in the paper, the algorithm used in sam2.20 is actually different from that of SAM. Currently, a new version (Version 3.0) is available. However, it seems that there is no change in the algorithm used in sam2.20 and Version 3.0 (pages 27–31 of [13]).

Recent studies have found that SAM does not control FDR well [14-16]. In one class case, it has been observed in [14] that the null scores of the DE genes generated from the one class version of the SAM statistic (1) are more dispersed than the null scores from the equivalently expressed (EE) genes. Such over-dispersion may lead to over-estimation of FDR. In the two experimental condition comparisons as we are considering in this paper, the same over-dispersion problem was also observed and discussed in [12,15,17,18]. Larson et al. [19] argued for caution when using the Excel version of SAM. The over-dispersed null scores show that the SAM statistic when being used as the null statistic does not have the true null distribution. In addition to the over-dispersion problem, our study also found that the distinct feature of SAM: the use of the displacement between the ordered test statistics and the expected null scores to look for the cutoffs, may lead to biased and even conflicting results when the DE and EE genes are not well separated.

The above mentioned problems of SAM also apply to sam2.20. Nevertheless, SAM has one much worse problem: its method for estimating FDR, which we will show does not produce meaningful results. This method has been abandoned in sam2.20. The discrepancies between SAM and sam2.20, combined with their potential problems, are the motivation of this paper. The objective is to provide a thorough evaluation of SAM and sam2.20 and analyze the pitfalls of the two methods. Our findings show that the performance of SAM and sam2.20 can be improved by looking into the following two aspects: 1) using a null statistic which can generate the null scores which have the true null distribution of the SAM statistic; and 2) avoiding the use of the order statistics in search of the cutoffs. It turns out that the correction of Aspect 1 requires construction of totally different test and null statistics [20], which is beyond the scope of the current paper. For this reason, we will only focus on the correction of Aspect 2 in this paper. The paper is organized as follows. Section 2 gives a review of the algorithms of SAM and sam2.20. The discrepancies between them will be pointed out and the impacts of these discrepancies will be studied. In Section 3, we discuss the potential problems of SAM and sam2.20. In Section 4, we re-visit the conventional symmetric cutoff method. Simulations carried out in Section 5 show that the symmetric cutoff method has advantage over sam2.20 when the number of induced genes in the microarray data is not too different from that of repressed genes while sam2.20 is a better choice if there are overwhelmingly more induced genes than repressed genes (or vice versa). To overcome the over-estimation problem, we further propose a modified version of the FDR correction method [14]. For comparison, the same idea was also applied to sam2.20. Numerical results show that such modification provides improved control of FDR for both sam2.20 and the symmetric cutoff method. Finally, both methods were applied to the leukaemia data [21] to compare their performance.

## 2 Results

### 2.1 A review of SAM

Let *X*_*ij *_be the expression level of gene *i *under experimental condition 1 in the *j*th replicate and *Y*_*ik *_be the expression level of gene *i *under experimental condition 2 in the *k*th replicate, where *i *= 1, ..., *n*, *j *= 1, ..., *J*, and *k *= 1, ..., *K*. The SAM statistic is defined as follows:

d(i)=X¯i−Y¯is(i)+s0
 MathType@MTEF@5@5@+=feaafiart1ev1aaatCvAUfKttLearuWrP9MDH5MBPbIqV92AaeXatLxBI9gBaebbnrfifHhDYfgasaacH8akY=wiFfYdH8Gipec8Eeeu0xXdbba9frFj0=OqFfea0dXdd9vqai=hGuQ8kuc9pgc9s8qqaq=dirpe0xb9q8qiLsFr0=vr0=vr0dc8meaabaqaciaacaGaaeqabaqabeGadaaakeaacqWGKbazcqGGOaakcqWGPbqAcqGGPaqkcqGH9aqpdaWcaaqaaiqbdIfayzaaraWaaSbaaSqaaiabdMgaPbqabaGccqGHsislcuWGzbqwgaqeamaaBaaaleaacqWGPbqAaeqaaaGcbaGaem4CamNaeiikaGIaemyAaKMaeiykaKIaey4kaSIaem4Cam3aaSbaaSqaaiabicdaWaqabaaaaaaa@40BA@

where X¯i
 MathType@MTEF@5@5@+=feaafiart1ev1aaatCvAUfKttLearuWrP9MDH5MBPbIqV92AaeXatLxBI9gBaebbnrfifHhDYfgasaacH8akY=wiFfYdH8Gipec8Eeeu0xXdbba9frFj0=OqFfea0dXdd9vqai=hGuQ8kuc9pgc9s8qqaq=dirpe0xb9q8qiLsFr0=vr0=vr0dc8meaabaqaciaacaGaaeqabaqabeGadaaakeaacuWGybawgaqeamaaBaaaleaacqWGPbqAaeqaaaaa@2F84@ and Y¯i
 MathType@MTEF@5@5@+=feaafiart1ev1aaatCvAUfKttLearuWrP9MDH5MBPbIqV92AaeXatLxBI9gBaebbnrfifHhDYfgasaacH8akY=wiFfYdH8Gipec8Eeeu0xXdbba9frFj0=OqFfea0dXdd9vqai=hGuQ8kuc9pgc9s8qqaq=dirpe0xb9q8qiLsFr0=vr0=vr0dc8meaabaqaciaacaGaaeqabaqabeGadaaakeaacuWGzbqwgaqeamaaBaaaleaacqWGPbqAaeqaaaaa@2F86@ are the averages of expression levels for gene *i *under experimental conditions 1 and 2. The gene-specific scatter *s*(*i*) is defined as

s(i)=a{∑j=1J(Xij−X¯i)2+∑k=1K(Yij−Y¯i)2},
 MathType@MTEF@5@5@+=feaafiart1ev1aaatCvAUfKttLearuWrP9MDH5MBPbIqV92AaeXatLxBI9gBaebbnrfifHhDYfgasaacH8akY=wiFfYdH8Gipec8Eeeu0xXdbba9frFj0=OqFfea0dXdd9vqai=hGuQ8kuc9pgc9s8qqaq=dirpe0xb9q8qiLsFr0=vr0=vr0dc8meaabaqaciaacaGaaeqabaqabeGadaaakeaacqWGZbWCcqGGOaakcqWGPbqAcqGGPaqkcqGH9aqpdaGcaaqaaiabdggaHjabcUha7naaqahabaGaeiikaGIaemiwaG1aaSbaaSqaaiabdMgaPjabdQgaQbqabaGccqGHsislcuWGybawgaqeamaaBaaaleaacqWGPbqAaeqaaOGaeiykaKYaaWbaaSqabeaacqaIYaGmaaaabaGaemOAaOMaeyypa0JaeGymaedabaGaemOsaOeaniabggHiLdGccqGHRaWkdaaeWbqaaiabcIcaOiabdMfaznaaBaaaleaacqWGPbqAcqWGQbGAaeqaaOGaeyOeI0IafmywaKLbaebadaWgaaWcbaGaemyAaKgabeaakiabcMcaPmaaCaaaleqabaGaeGOmaidaaaqaaiabdUgaRjabg2da9iabigdaXaqaaiabdUealbqdcqGHris5aOGaeiyFa0haleqaaOGaeiilaWcaaa@5B58@

where *a *= (1/*J *+ 1/*K*)/(*J *+ *K *- 2). The constant *s*_0 _is chosen to minimize the coefficient of variation of *d*(*i*).

The null scores are obtained by permuting the pooled data {*X*_*i*1_, ..., *X*_*iJ*_; *Y*_*i*1_, ..., *Y*_*iK*_} and then treating the first *J *expression levels as the observations under experimental condition 1 and the remaining *K *as the observations under experimental condition 2. For a particular permutation *b*, denote the permuted data by {Xijb}j=1J
 MathType@MTEF@5@5@+=feaafiart1ev1aaatCvAUfKttLearuWrP9MDH5MBPbIqV92AaeXatLxBI9gBaebbnrfifHhDYfgasaacH8akY=wiFfYdH8Gipec8Eeeu0xXdbba9frFj0=OqFfea0dXdd9vqai=hGuQ8kuc9pgc9s8qqaq=dirpe0xb9q8qiLsFr0=vr0=vr0dc8meaabaqaciaacaGaaeqabaqabeGadaaakeaacqGG7bWEcqWGybawdaqhaaWcbaGaemyAaKMaemOAaOgabaGaemOyaigaaOGaeiyFa03aa0baaSqaaiabdQgaQjabg2da9iabigdaXaqaaiabdQeakbaaaaa@39BE@ and {Yikb}k=1K
 MathType@MTEF@5@5@+=feaafiart1ev1aaatCvAUfKttLearuWrP9MDH5MBPbIqV92AaeXatLxBI9gBaebbnrfifHhDYfgasaacH8akY=wiFfYdH8Gipec8Eeeu0xXdbba9frFj0=OqFfea0dXdd9vqai=hGuQ8kuc9pgc9s8qqaq=dirpe0xb9q8qiLsFr0=vr0=vr0dc8meaabaqaciaacaGaaeqabaqabeGadaaakeaacqGG7bWEcqWGzbqwdaqhaaWcbaGaemyAaKMaem4AaSgabaGaemOyaigaaOGaeiyFa03aa0baaSqaaiabdUgaRjabg2da9iabigdaXaqaaiabdUealbaaaaa@39C6@.

Then, the null scores, *d*^*b*^(*i*), *i *= 1, ..., *n*, for permutation *b *are calculated from the following formula:

db(i)=X¯ib−Y¯ibs(i)b+s0,
 MathType@MTEF@5@5@+=feaafiart1ev1aaatCvAUfKttLearuWrP9MDH5MBPbIqV92AaeXatLxBI9gBaebbnrfifHhDYfgasaacH8akY=wiFfYdH8Gipec8Eeeu0xXdbba9frFj0=OqFfea0dXdd9vqai=hGuQ8kuc9pgc9s8qqaq=dirpe0xb9q8qiLsFr0=vr0=vr0dc8meaabaqaciaacaGaaeqabaqabeGadaaakeaacqWGKbazdaahaaWcbeqaaiabdkgaIbaakiabcIcaOiabdMgaPjabcMcaPiabg2da9maalaaabaGafmiwaGLbaebadaqhaaWcbaGaemyAaKgabaGaemOyaigaaOGaeyOeI0IafmywaKLbaebadaqhaaWcbaGaemyAaKgabaGaemOyaigaaaGcbaGaem4CamNaeiikaGIaemyAaKMaeiykaKYaaWbaaSqabeaacqWGIbGyaaGccqGHRaWkcqWGZbWCdaWgaaWcbaGaeGimaadabeaaaaGccqGGSaalaaa@4748@

where X¯ib
 MathType@MTEF@5@5@+=feaafiart1ev1aaatCvAUfKttLearuWrP9MDH5MBPbIqV92AaeXatLxBI9gBaebbnrfifHhDYfgasaacH8akY=wiFfYdH8Gipec8Eeeu0xXdbba9frFj0=OqFfea0dXdd9vqai=hGuQ8kuc9pgc9s8qqaq=dirpe0xb9q8qiLsFr0=vr0=vr0dc8meaabaqaciaacaGaaeqabaqabeGadaaakeaacuWGybawgaqeamaaDaaaleaacqWGPbqAaeaacqWGIbGyaaaaaa@30D2@ is the sample mean of {Xijb}j=1J
 MathType@MTEF@5@5@+=feaafiart1ev1aaatCvAUfKttLearuWrP9MDH5MBPbIqV92AaeXatLxBI9gBaebbnrfifHhDYfgasaacH8akY=wiFfYdH8Gipec8Eeeu0xXdbba9frFj0=OqFfea0dXdd9vqai=hGuQ8kuc9pgc9s8qqaq=dirpe0xb9q8qiLsFr0=vr0=vr0dc8meaabaqaciaacaGaaeqabaqabeGadaaakeaacqGG7bWEcqWGybawdaqhaaWcbaGaemyAaKMaemOAaOgabaGaemOyaigaaOGaeiyFa03aa0baaSqaaiabdQgaQjabg2da9iabigdaXaqaaiabdQeakbaaaaa@39BE@, Y¯ib
 MathType@MTEF@5@5@+=feaafiart1ev1aaatCvAUfKttLearuWrP9MDH5MBPbIqV92AaeXatLxBI9gBaebbnrfifHhDYfgasaacH8akY=wiFfYdH8Gipec8Eeeu0xXdbba9frFj0=OqFfea0dXdd9vqai=hGuQ8kuc9pgc9s8qqaq=dirpe0xb9q8qiLsFr0=vr0=vr0dc8meaabaqaciaacaGaaeqabaqabeGadaaakeaacuWGzbqwgaqeamaaDaaaleaacqWGPbqAaeaacqWGIbGyaaaaaa@30D4@ is the sample mean of {Yikb}k=1K
 MathType@MTEF@5@5@+=feaafiart1ev1aaatCvAUfKttLearuWrP9MDH5MBPbIqV92AaeXatLxBI9gBaebbnrfifHhDYfgasaacH8akY=wiFfYdH8Gipec8Eeeu0xXdbba9frFj0=OqFfea0dXdd9vqai=hGuQ8kuc9pgc9s8qqaq=dirpe0xb9q8qiLsFr0=vr0=vr0dc8meaabaqaciaacaGaaeqabaqabeGadaaakeaacqGG7bWEcqWGzbqwdaqhaaWcbaGaemyAaKMaem4AaSgabaGaemOyaigaaOGaeiyFa03aa0baaSqaaiabdUgaRjabg2da9iabigdaXaqaaiabdUealbaaaaa@39C6@, and the gene-specific scatter is calculated by

sb(i)=a{∑j=1J(Xijb−X¯ib)2+∑k=1K(Yijb−Y¯ib)2}.
 MathType@MTEF@5@5@+=feaafiart1ev1aaatCvAUfKttLearuWrP9MDH5MBPbIqV92AaeXatLxBI9gBaebbnrfifHhDYfgasaacH8akY=wiFfYdH8Gipec8Eeeu0xXdbba9frFj0=OqFfea0dXdd9vqai=hGuQ8kuc9pgc9s8qqaq=dirpe0xb9q8qiLsFr0=vr0=vr0dc8meaabaqaciaacaGaaeqabaqabeGadaaakeaacqWGZbWCdaahaaWcbeqaaiabdkgaIbaakiabcIcaOiabdMgaPjabcMcaPiabg2da9maakaaabaGaemyyaeMaei4EaS3aaabCaeaacqGGOaakcqWGybawdaqhaaWcbaGaemyAaKMaemOAaOgabaGaemOyaigaaOGaeyOeI0IafmiwaGLbaebadaqhaaWcbaGaemyAaKgabaGaemOyaigaaOGaeiykaKYaaWbaaSqabeaacqaIYaGmaaaabaGaemOAaOMaeyypa0JaeGymaedabaGaemOsaOeaniabggHiLdGccqGHRaWkdaaeWbqaaiabcIcaOiabdMfaznaaDaaaleaacqWGPbqAcqWGQbGAaeaacqWGIbGyaaGccqGHsislcuWGzbqwgaqeamaaDaaaleaacqWGPbqAaeaacqWGIbGyaaGccqGGPaqkdaahaaWcbeqaaiabikdaYaaaaeaacqWGRbWAcqGH9aqpcqaIXaqmaeaacqWGlbWsa0GaeyyeIuoakiabc2ha9bWcbeaakiabc6caUaaa@6218@

The SAM algorithm proposed in [9] can be stated as follows:

(a) Order the test statistics *d*(*i*), *i *= 1, ..., *n *according to their magnitudes as *d*_(1) _≤ *d*_(2) _≤ ⋯ ≤ *d*_(*n*)_.

(b) For each permutation *b*, compute the ordered null scores, and denote them by *d*_(1)_(*b*) ≤ *d*_(2)_(*b*) ≤ ⋯ ≤ *d*_(*n*)_(*b*), *b *= 1, ..., *B*, where *B *is the total number of permutations used.

(c) Calculate the expected null scores by d¯(i)=∑b=1Bd(i)(b)/B
 MathType@MTEF@5@5@+=feaafiart1ev1aaatCvAUfKttLearuWrP9MDH5MBPbIqV92AaeXatLxBI9gBaebbnrfifHhDYfgasaacH8akY=wiFfYdH8Gipec8Eeeu0xXdbba9frFj0=OqFfea0dXdd9vqai=hGuQ8kuc9pgc9s8qqaq=dirpe0xb9q8qiLsFr0=vr0=vr0dc8meaabaqaciaacaGaaeqabaqabeGadaaakeaacuWGKbazgaqeamaaBaaaleaacqGGOaakcqWGPbqAcqGGPaqkaeqaaOGaeyypa0ZaaabCaeaacqWGKbazdaWgaaWcbaGaeiikaGIaemyAaKMaeiykaKcabeaakiabcIcaOiabdkgaIjabcMcaPiabc+caViabdkeacbWcbaGaemOyaiMaeyypa0JaeGymaedabaGaemOqaieaniabggHiLdaaaa@4276@.

(d) Plot the ordered test statistics *d*_(1)_, *d*_(2)_, ⋯, *d*_(*n*) _against the expected null scores d¯(1),d¯(2),⋯,d¯(n)
 MathType@MTEF@5@5@+=feaafiart1ev1aaatCvAUfKttLearuWrP9MDH5MBPbIqV92AaeXatLxBI9gBaebbnrfifHhDYfgasaacH8akY=wiFfYdH8Gipec8Eeeu0xXdbba9frFj0=OqFfea0dXdd9vqai=hGuQ8kuc9pgc9s8qqaq=dirpe0xb9q8qiLsFr0=vr0=vr0dc8meaabaqaciaacaGaaeqabaqabeGadaaakeaacuWGKbazgaqeamaaBaaaleaacqGGOaakcqaIXaqmcqGGPaqkaeqaaOGaeiilaWIafmizaqMbaebadaWgaaWcbaGaeiikaGIaeGOmaiJaeiykaKcabeaakiabcYcaSiabl+UimjabcYcaSiqbdsgaKzaaraWaaSbaaSqaaiabcIcaOiabd6gaUjabcMcaPaqabaaaaa@3E6A@.

(e) For each possible threshold Δ, a gene is called significant if

|d(i)−d¯(i)|>Δ.
 MathType@MTEF@5@5@+=feaafiart1ev1aaatCvAUfKttLearuWrP9MDH5MBPbIqV92AaeXatLxBI9gBaebbnrfifHhDYfgasaacH8akY=wiFfYdH8Gipec8Eeeu0xXdbba9frFj0=OqFfea0dXdd9vqai=hGuQ8kuc9pgc9s8qqaq=dirpe0xb9q8qiLsFr0=vr0=vr0dc8meaabaqaciaacaGaaeqabaqabeGadaaakeaadaabdaqaaiabdsgaKnaaBaaaleaacqGGOaakcqWGPbqAcqGGPaqkaeqaaOGaeyOeI0IafmizaqMbaebadaWgaaWcbaGaeiikaGIaemyAaKMaeiykaKcabeaaaOGaay5bSlaawIa7aiabg6da+iabfs5aejabc6caUaaa@3D4D@

Then, the total number of genes declared significant is

TP_=#{i:|d(i)−d¯(i)|>Δ}.
 MathType@MTEF@5@5@+=feaafiart1ev1aaatCvAUfKttLearuWrP9MDH5MBPbIqV92AaeXatLxBI9gBaebbnrfifHhDYfgasaacH8akY=wiFfYdH8Gipec8Eeeu0xXdbba9frFj0=OqFfea0dXdd9vqai=hGuQ8kuc9pgc9s8qqaq=dirpe0xb9q8qiLsFr0=vr0=vr0dc8meaabaqaciaacaGaaeqabaqabeGadaaakeaadaqiaaqaaiabdsfaujabdcfaqbGaayPadaGaeyypa0Jaei4iamIaei4EaSNaemyAaKMaeiOoaOZaaqWaaeaacqWGKbazdaWgaaWcbaGaeiikaGIaemyAaKMaeiykaKcabeaakiabgkHiTiqbdsgaKzaaraWaaSbaaSqaaiabcIcaOiabdMgaPjabcMcaPaqabaaakiaawEa7caGLiWoacqGH+aGpcqqHuoarcqGG9bqFcqGGUaGlaaa@4794@

(f) Denote the set of significant genes declared in (e) by *T*. The FDR is estimated by FDR_=FP_/TP_
 MathType@MTEF@5@5@+=feaafiart1ev1aaatCvAUfKttLearuWrP9MDH5MBPbIqV92AaeXatLxBI9gBaebbnrfifHhDYfgasaacH8akY=wiFfYdH8Gipec8Eeeu0xXdbba9frFj0=OqFfea0dXdd9vqai=hGuQ8kuc9pgc9s8qqaq=dirpe0xb9q8qiLsFr0=vr0=vr0dc8meaabaqaciaacaGaaeqabaqabeGadaaakeaadaqiaaqaaiabdAeagjabdseaejabdkfasbGaayPadaGaeyypa0ZaaecaaeaacqWGgbGrcqWGqbauaiaawkWaaiabc+caVmaaHaaabaGaemivaqLaemiuaafacaGLcmaaaaa@38C9@, where

FP_=∑b=1B#{i∈T:db(i)>δU or db(i)<δL}/B.
 MathType@MTEF@5@5@+=feaafiart1ev1aaatCvAUfKttLearuWrP9MDH5MBPbIqV92AaeXatLxBI9gBaebbnrfifHhDYfgasaacH8akY=wiFfYdH8Gipec8Eeeu0xXdbba9frFj0=OqFfea0dXdd9vqai=hGuQ8kuc9pgc9s8qqaq=dirpe0xb9q8qiLsFr0=vr0=vr0dc8meaabaqaciaacaGaaeqabaqabeGadaaakeaadaqiaaqaaiabdAeagjabdcfaqbGaayPadaGaeyypa0ZaaabCaeaacqGGJaWicqGG7bWEcqWGPbqAcqGHiiIZcqWGubavcqGG6aGocqWGKbazdaahaaWcbeqaaiabdkgaIbaakiabcIcaOiabdMgaPjabcMcaPiabg6da+GGaciab=r7aKnaaBaaaleaacqWGvbqvaeqaaaqaaiabdkgaIjabg2da9iabigdaXaqaaiabdkeacbqdcqGHris5aOGaeeiiaaIaee4Ba8MaeeOCaiNaeeiiaaIaemizaq2aaWbaaSqabeaacqWGIbGyaaGccqGGOaakcqWGPbqAcqGGPaqkcqGH8aapcqWF0oazdaWgaaWcbaGaemitaWeabeaakiabc2ha9jabc+caViabdkeacjabc6caUaaa@5B24@

The two values *δ*_*U*_, *δ*_*L *_used in (6) are the horizontal cutoffs. They are defined as the smallest *d*_(*i*) _among the significant positive genes and the largest *d*_(*i*) _among the significant negative genes.

The above SAM algorithm was also illustrated in [10] except that they proposed to estimate the number of FP by:

FP_=∑b=1B#{i:|d(i)(b)−d¯(i,b)|>Δ}/B,
 MathType@MTEF@5@5@+=feaafiart1ev1aaatCvAUfKttLearuWrP9MDH5MBPbIqV92AaeXatLxBI9gBaebbnrfifHhDYfgasaacH8akY=wiFfYdH8Gipec8Eeeu0xXdbba9frFj0=OqFfea0dXdd9vqai=hGuQ8kuc9pgc9s8qqaq=dirpe0xb9q8qiLsFr0=vr0=vr0dc8meaabaqaciaacaGaaeqabaqabeGadaaakeaadaqiaaqaaiabdAeagjabdcfaqbGaayPadaGaeyypa0ZaaabCaeaacqGGJaWicqGG7bWEcqWGPbqAcqGG6aGodaabdaqaaiabdsgaKnaaBaaaleaacqGGOaakcqWGPbqAcqGGPaqkaeqaaOGaeiikaGIaemOyaiMaeiykaKIaeyOeI0IafmizaqMbaebadaWgaaWcbaGaeiikaGIaemyAaKMaeiilaWIaemOyaiMaeiykaKcabeaaaOGaay5bSlaawIa7aiabg6da+iabfs5aejabc2ha9jabc+caViabdkeacbWcbaGaemOyaiMaeyypa0JaeGymaedabaGaemOqaieaniabggHiLdGccqGGSaalaaa@552F@

where d¯(i,b)=∑c≠bd(i)(c)/(B−1)
 MathType@MTEF@5@5@+=feaafiart1ev1aaatCvAUfKttLearuWrP9MDH5MBPbIqV92AaeXatLxBI9gBaebbnrfifHhDYfgasaacH8akY=wiFfYdH8Gipec8Eeeu0xXdbba9frFj0=OqFfea0dXdd9vqai=hGuQ8kuc9pgc9s8qqaq=dirpe0xb9q8qiLsFr0=vr0=vr0dc8meaabaqaciaacaGaaeqabaqabeGadaaakeaacuWGKbazgaqeamaaBaaaleaacqGGOaakcqWGPbqAcqGGSaalcqWGIbGycqGGPaqkaeqaaOGaeyypa0ZaaabuaeaacqWGKbazdaWgaaWcbaGaeiikaGIaemyAaKMaeiykaKcabeaakiabcIcaOiabdogaJjabcMcaPiabc+caViabcIcaOiabdkeacjabgkHiTiabigdaXiabcMcaPaWcbaGaem4yamMaeyiyIKRaemOyaigabeqdcqGHris5aaaa@4828@.

### 2.2 A review of sam2.20

Comparing the above SAM algorithm with that used in sam2.20 [13], we see that there are two differences on Steps (e) and (f). The difference on (e) is on how a gene is declared significant. For a fixed threshold Δ, starting at the origin, and moving up to the right find the first *i *= *i*_1 _such that *d*_(*i*) _- d¯(i)
 MathType@MTEF@5@5@+=feaafiart1ev1aaatCvAUfKttLearuWrP9MDH5MBPbIqV92AaeXatLxBI9gBaebbnrfifHhDYfgasaacH8akY=wiFfYdH8Gipec8Eeeu0xXdbba9frFj0=OqFfea0dXdd9vqai=hGuQ8kuc9pgc9s8qqaq=dirpe0xb9q8qiLsFr0=vr0=vr0dc8meaabaqaciaacaGaaeqabaqabeGadaaakeaacuWGKbazgaqeamaaBaaaleaacqGGOaakcqWGPbqAcqGGPaqkaeqaaaaa@314E@ > Δ. All genes past *i*_1 _are called "significantly positive". Similarly, starting from the origin, move down to the left and find the first *i *= *i*_2 _such that *d*_(*i*) _- d¯(i)
 MathType@MTEF@5@5@+=feaafiart1ev1aaatCvAUfKttLearuWrP9MDH5MBPbIqV92AaeXatLxBI9gBaebbnrfifHhDYfgasaacH8akY=wiFfYdH8Gipec8Eeeu0xXdbba9frFj0=OqFfea0dXdd9vqai=hGuQ8kuc9pgc9s8qqaq=dirpe0xb9q8qiLsFr0=vr0=vr0dc8meaabaqaciaacaGaaeqabaqabeGadaaakeaacuWGKbazgaqeamaaBaaaleaacqGGOaakcqWGPbqAcqGGPaqkaeqaaaaa@314E@ < -Δ. All genes past *i*_2 _are called "significant negative"; see Steps 6 and 7 on Page 28 of [13]. Denote *δ*_*L *_= d(i2)
 MathType@MTEF@5@5@+=feaafiart1ev1aaatCvAUfKttLearuWrP9MDH5MBPbIqV92AaeXatLxBI9gBaebbnrfifHhDYfgasaacH8akY=wiFfYdH8Gipec8Eeeu0xXdbba9frFj0=OqFfea0dXdd9vqai=hGuQ8kuc9pgc9s8qqaq=dirpe0xb9q8qiLsFr0=vr0=vr0dc8meaabaqaciaacaGaaeqabaqabeGadaaakeaacqWGKbazdaWgaaWcbaGaeiikaGIaemyAaK2aaSbaaWqaaiabikdaYaqabaWccqGGPaqkaeqaaaaa@3260@, *δ*_*U *_= d(i1)
 MathType@MTEF@5@5@+=feaafiart1ev1aaatCvAUfKttLearuWrP9MDH5MBPbIqV92AaeXatLxBI9gBaebbnrfifHhDYfgasaacH8akY=wiFfYdH8Gipec8Eeeu0xXdbba9frFj0=OqFfea0dXdd9vqai=hGuQ8kuc9pgc9s8qqaq=dirpe0xb9q8qiLsFr0=vr0=vr0dc8meaabaqaciaacaGaaeqabaqabeGadaaakeaacqWGKbazdaWgaaWcbaGaeiikaGIaemyAaK2aaSbaaWqaaiabigdaXaqabaWccqGGPaqkaeqaaaaa@325E@. This process can be expressed as

(e') For each possible threshold Δ, a gene is called significant positive if *d*(*i*) > *δ*_*U*_, or significant negative if *d*(*i*) <*δ*_*L*_.

The total number of genes declared significant is

TP_
 MathType@MTEF@5@5@+=feaafiart1ev1aaatCvAUfKttLearuWrP9MDH5MBPbIqV92AaeXatLxBI9gBaebbnrfifHhDYfgasaacH8akY=wiFfYdH8Gipec8Eeeu0xXdbba9frFj0=OqFfea0dXdd9vqai=hGuQ8kuc9pgc9s8qqaq=dirpe0xb9q8qiLsFr0=vr0=vr0dc8meaabaqaciaacaGaaeqabaqabeGadaaakeaadaqiaaqaaiabdsfaujabdcfaqbGaayPadaaaaa@2FC8@ = #{1 ≤ *i *≤ *n*: *d*(*i*) > *δ*_*U *_or *d*(*i*) <*δ*_*L*_}.

The difference on Step (f) is in the estimation of FP and FDR. Note that SAM estimates the FP only using the null scores from the genes called significant in Step (e). sam2.20 uses the null scores from all the genes to estimate the FP:

(f') FP_=median(FP_(1),⋯,FP_(B)),
 MathType@MTEF@5@5@+=feaafiart1ev1aaatCvAUfKttLearuWrP9MDH5MBPbIqV92AaeXatLxBI9gBaebbnrfifHhDYfgasaacH8akY=wiFfYdH8Gipec8Eeeu0xXdbba9frFj0=OqFfea0dXdd9vqai=hGuQ8kuc9pgc9s8qqaq=dirpe0xb9q8qiLsFr0=vr0=vr0dc8meaabaqaciaacaGaaeqabaqabeGadaaakeaacqGGOaakcqqGMbGzcqGGNaWjcqGGPaqkcqqGGaaidaqiaaqaaiabdAeagjabdcfaqbGaayPadaGaeyypa0JaeeyBa0MaeeyzauMaeeizaqMaeeyAaKMaeeyyaeMaeeOBa42aaeWaaeaadaqiaaqaaiabdAeagjabdcfaqbGaayPadaGaeiikaGIaeGymaeJaeiykaKIaeiilaWIaeS47IWKaeiilaWYaaecaaeaacqWGgbGrcqWGqbauaiaawkWaaiabcIcaOiabdkeacjabcMcaPaGaayjkaiaawMcaaiabcYcaSaaa@4ED2@

where FP_
 MathType@MTEF@5@5@+=feaafiart1ev1aaatCvAUfKttLearuWrP9MDH5MBPbIqV92AaeXatLxBI9gBaebbnrfifHhDYfgasaacH8akY=wiFfYdH8Gipec8Eeeu0xXdbba9frFj0=OqFfea0dXdd9vqai=hGuQ8kuc9pgc9s8qqaq=dirpe0xb9q8qiLsFr0=vr0=vr0dc8meaabaqaciaacaGaaeqabaqabeGadaaakeaadaqiaaqaaiabdAeagjabdcfaqbGaayPadaaaaa@2FAC@(*b*) = #{1 ≤ *i *≤ *n*: *d*^*b*^(*i*) > *δ*_*U *_or *d*^*b*^(*i*) <*δ*_*L*_, *b *= 1, ..., *B*. Subsequently, the FDR is estimated by FDR_=π^0FP_/TP_
 MathType@MTEF@5@5@+=feaafiart1ev1aaatCvAUfKttLearuWrP9MDH5MBPbIqV92AaeXatLxBI9gBaebbnrfifHhDYfgasaacH8akY=wiFfYdH8Gipec8Eeeu0xXdbba9frFj0=OqFfea0dXdd9vqai=hGuQ8kuc9pgc9s8qqaq=dirpe0xb9q8qiLsFr0=vr0=vr0dc8meaabaqaciaacaGaaeqabaqabeGadaaakeaadaqiaaqaaiabdAeagjabdseaejabdkfasbGaayPadaGaeyypa0dcciGaf8hWdaNbaKaadaWgaaWcbaGaeGimaadabeaakmaaHaaabaGaemOrayKaemiuaafacaGLcmaacqGGVaWldaqiaaqaaiabdsfaujabdcfaqbGaayPadaaaaa@3BC1@, where π^0
 MathType@MTEF@5@5@+=feaafiart1ev1aaatCvAUfKttLearuWrP9MDH5MBPbIqV92AaeXatLxBI9gBaebbnrfifHhDYfgasaacH8akY=wiFfYdH8Gipec8Eeeu0xXdbba9frFj0=OqFfea0dXdd9vqai=hGuQ8kuc9pgc9s8qqaq=dirpe0xb9q8qiLsFr0=vr0=vr0dc8meaabaqaciaacaGaaeqabaqabeGadaaakeaaiiGacuWFapaCgaqcamaaBaaaleaacqaIWaamaeqaaaaa@2F9A@ is the estimated proportion of non-DE genes. A natural spline based estimator π^0
 MathType@MTEF@5@5@+=feaafiart1ev1aaatCvAUfKttLearuWrP9MDH5MBPbIqV92AaeXatLxBI9gBaebbnrfifHhDYfgasaacH8akY=wiFfYdH8Gipec8Eeeu0xXdbba9frFj0=OqFfea0dXdd9vqai=hGuQ8kuc9pgc9s8qqaq=dirpe0xb9q8qiLsFr0=vr0=vr0dc8meaabaqaciaacaGaaeqabaqabeGadaaakeaaiiGacuWFapaCgaqcamaaBaaaleaacqaIWaamaeqaaaaa@2F9A@ is used in sam2.20 [22].

### 2.3 The impact of the change of algorithms

#### 2.3.1 The impact of the difference between Step (e) of SAM and Step (e') of sam2.20

In Step (e) of SAM, only those genes with displacement from d¯(i)
 MathType@MTEF@5@5@+=feaafiart1ev1aaatCvAUfKttLearuWrP9MDH5MBPbIqV92AaeXatLxBI9gBaebbnrfifHhDYfgasaacH8akY=wiFfYdH8Gipec8Eeeu0xXdbba9frFj0=OqFfea0dXdd9vqai=hGuQ8kuc9pgc9s8qqaq=dirpe0xb9q8qiLsFr0=vr0=vr0dc8meaabaqaciaacaGaaeqabaqabeGadaaakeaacuWGKbazgaqeamaaBaaaleaacqGGOaakcqWGPbqAcqGGPaqkaeqaaaaa@314E@ larger than Δ are called significant. This means that, if gene *i *is called significant positive (or significant negative), it does not imply that gene *j *with *d*(*j*) > *d*(*i*) (resp. *d*(*j*) <*d*(*i*)) will be called significant as well. Because of this, it is claimed in [9] that the genes identified as significant by SAM do not necessarily have the largest relative changes in gene expression. To better understand how SAM and sam2.20 work differently, we carried out the following simulation. In the simulation, the data were generated from the following model:

*X*_*ij *_= *μ*_*i *_+ *ε*_*ij *_and *Y*_*ik *_= *η*_i _+ *ω*_*ik *_for *i *= 1, ..., *n*, *j *= 1, ..., *J*, *k *= 1, ..., *K*,

where *n *= 5000, *J *= *K *= 4 and *ε*_*ij *_and *ω*_*ik *_are the i.i.d. random errors from *N*(0,1). For the first 100 genes, *μ*_*i *_= 0 and *η*_*i *_~ *N*(1,1), and for the last 100 genes, *μ*_*i *_= 0 and *η*_*i *_~ *N*(-1,1). The middle 4800 genes were generated with *μ*_*i *_= *η*_*i *_= 0. Hence, there are in total 200 differentially expressed genes.

**Figure 1:**
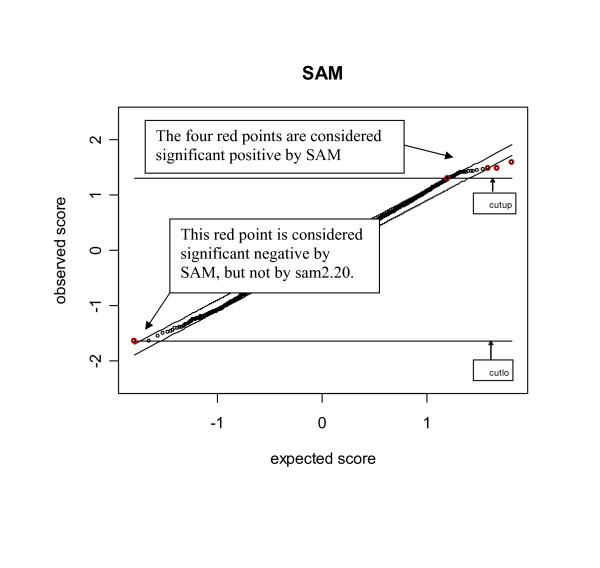
**The SAM plot obtained by using the SAM algorithm**. The red points are the points declared significant by SAM. The two horizontal lines refer to the lower cutoff *δ*_*L *_(=cutlo) and the upper cutoff *δ*_*U *_(=cutlup) from SAM. The threshold used is Δ = 0.099.

Figure 1 reports the findings from SAM. In Figure 1, the points in red are the genes declared significant by SAM. There are in total 5 points with displacement larger than Δ, of which 4 are called significant positive and 1 is called significant negative. The cutoff *δ*_*L*_(= -1.649701) is the value of the test statistic of the only significant negative gene, and *δ*_*U*_(= 1.3068) is the minimum value of the test statistics of the 4 significant positive genes. It can be clearly seen from Figure 1 that many points (black dots) beyond the horizontal cutoffs are not called significant by SAM. The reason is that these points, although having test statistics of greater magnitudes than the relevant cutoff, do not have displacement larger than the threshold Δ.

**Figure 2:**
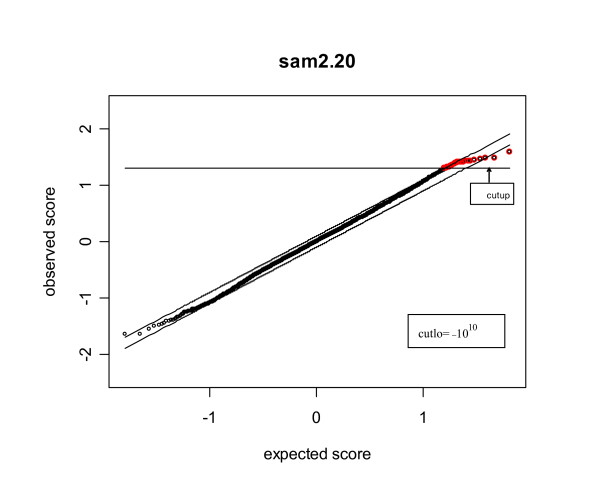
**The sam plot obtained from sam2.20**. The red points are the points declared significant by sam2.20. The horizontal line refers to the upper cutoff *δ*_*U *_(=cutlup) from sam2.20. The horizontal line corresponding to the lower cutoff *δ*_*L *_(=cutlo) does not show up in the plot since *δ*_*L *_= -10^10^. The threshold Δ used is the same as that used in producing Figure 1.

However, this feature has been changed in the algorithm used in sam2.20 due to the use of Step (e'). Figure 2 is the plot obtained from sam2.20 under the same setup as that of Figure 1. By checking Figure 2, we see that two changes have happened. The first change is the cutoffs. Note that the cutoffs from SAM are -1.6497 and 1.3068, respectively. Nevertheless, Figure 2 shows that the cutoffs from sam2.20 have become -10^10 ^and 1.3068. The lower cutoff was arbitrarily set at -10^10^ since the only point having displacement greater than does not satisfy *d*_(*i*) _- d¯(i)
 MathType@MTEF@5@5@+=feaafiart1ev1aaatCvAUfKttLearuWrP9MDH5MBPbIqV92AaeXatLxBI9gBaebbnrfifHhDYfgasaacH8akY=wiFfYdH8Gipec8Eeeu0xXdbba9frFj0=OqFfea0dXdd9vqai=hGuQ8kuc9pgc9s8qqaq=dirpe0xb9q8qiLsFr0=vr0=vr0dc8meaabaqaciaacaGaaeqabaqabeGadaaakeaacuWGKbazgaqeamaaBaaaleaacqGGOaakcqWGPbqAcqGGPaqkaeqaaaaa@314E@ < -Δ. The second change is the number of significant genes. Step (e') of sam2.20 declares all the genes with the test statistics exceeding the cutoffs as significant. Since there are in total 24 points exceeding the upper cutoff *δ*_*U *_= 1.3068, sam2.20 declares all these 24 points as significant positive. Note that no point in Figure 2 is declared significant negative since there is no point with value below the lower cutoff -10^10^.

#### 2.3.2 The impact of the difference between Steps (f) of SAM and Step (f') of sam2.20

The change from Step (f) of SAM to Step (f') of sam2.20 is a desirable change. The problem with Step (f) of SAM is that it only uses the genes identified as significant to estimate the number of FP. Although in the definition of FDR the number of FP refers to those among the genes declared significant, SAM ignored the fact that the FP genes among the significant genes are actually the genes which are falsely identified as significant among all the EE genes in the experiment. Hence, Step (f) of SAM which only calculates the number of FP among the genes called significant would severely under-estimate the true number of FP. However, note that Step (f') of sam2.20 actually uses all the genes instead of only those non-DE ones to estimate the FDR. This would certainly lead to over-estimation if no adjustment is done. As a result, the ratio FP_
 MathType@MTEF@5@5@+=feaafiart1ev1aaatCvAUfKttLearuWrP9MDH5MBPbIqV92AaeXatLxBI9gBaebbnrfifHhDYfgasaacH8akY=wiFfYdH8Gipec8Eeeu0xXdbba9frFj0=OqFfea0dXdd9vqai=hGuQ8kuc9pgc9s8qqaq=dirpe0xb9q8qiLsFr0=vr0=vr0dc8meaabaqaciaacaGaaeqabaqabeGadaaakeaadaqiaaqaaiabdAeagjabdcfaqbGaayPadaaaaa@2FAC@/TP_
 MathType@MTEF@5@5@+=feaafiart1ev1aaatCvAUfKttLearuWrP9MDH5MBPbIqV92AaeXatLxBI9gBaebbnrfifHhDYfgasaacH8akY=wiFfYdH8Gipec8Eeeu0xXdbba9frFj0=OqFfea0dXdd9vqai=hGuQ8kuc9pgc9s8qqaq=dirpe0xb9q8qiLsFr0=vr0=vr0dc8meaabaqaciaacaGaaeqabaqabeGadaaakeaadaqiaaqaaiabdsfaujabdcfaqbGaayPadaaaaa@2FC8@ is multiplied by a factor of π^0
 MathType@MTEF@5@5@+=feaafiart1ev1aaatCvAUfKttLearuWrP9MDH5MBPbIqV92AaeXatLxBI9gBaebbnrfifHhDYfgasaacH8akY=wiFfYdH8Gipec8Eeeu0xXdbba9frFj0=OqFfea0dXdd9vqai=hGuQ8kuc9pgc9s8qqaq=dirpe0xb9q8qiLsFr0=vr0=vr0dc8meaabaqaciaacaGaaeqabaqabeGadaaakeaaiiGacuWFapaCgaqcamaaBaaaleaacqaIWaamaeqaaaaa@2F9A@ to provide a reasonable estimate of FDR.

## 3 Potential problems of SAM and sam2.20

### 3.1 SAM's use of different standards to declare significance and its poor estimation of FDR

In addition to showing the difference between SAM and sam2.20, Figures 1 and 2 actually raise concerns about the use of SAM and sam2.20 in practice. Figure 1 shows that there are genes with test statistics exceeding *δ*_*L *_and *δ*_*U *_which are not identified as significant by Step (e) of SAM since they do not have displacement larger than the threshold Δ. However, Step (f) of SAM shows that such genes are considered as significant in the estimation of FDR. Hence, SAM used different standards to declare significance. The reason for SAM's use of different standards can be explained by the results of a simulation described as follows. The data used in the simulation were generated from model (10) under the same setup as that used in producing Figures 1 and 2, except that we used *μ*_*i *_= 0 and *η*_*i *_~ *N*(3,1) for the first 100 genes, and *μ*_*i *_= 0 and *η*_*i *_~ *N*(-3,1) for the last 100 genes.

Table 1 reports the results obtained from 100 simulations under the above described setup. Column 1 reports the average number of genes called significant by sam2.20 from 100 simulations. Column 2 reports the average number of true FP among the genes declared significant in each simulation. Columns 3–5 report the mean of estimated numbers of FP from SAM, (7) and sam2.20. Note that (7) uses the same rule as Step (e) of SAM to declare significance. The results from (7) should reflect what would happen if SAM had used the same standard (4) to declare significance for both test and null scores. It can be seen from Table 1 that (7) under-estimates the numbers of true FP significantly except in the last case (see the bottom row of Table 1). Among the 3 different mean values of TP_
 MathType@MTEF@5@5@+=feaafiart1ev1aaatCvAUfKttLearuWrP9MDH5MBPbIqV92AaeXatLxBI9gBaebbnrfifHhDYfgasaacH8akY=wiFfYdH8Gipec8Eeeu0xXdbba9frFj0=OqFfea0dXdd9vqai=hGuQ8kuc9pgc9s8qqaq=dirpe0xb9q8qiLsFr0=vr0=vr0dc8meaabaqaciaacaGaaeqabaqabeGadaaakeaadaqiaaqaaiabdsfaujabdcfaqbGaayPadaaaaa@2FC8@ reported in Table 1, the most relevant one is probably the case when mean TP_
 MathType@MTEF@5@5@+=feaafiart1ev1aaatCvAUfKttLearuWrP9MDH5MBPbIqV92AaeXatLxBI9gBaebbnrfifHhDYfgasaacH8akY=wiFfYdH8Gipec8Eeeu0xXdbba9frFj0=OqFfea0dXdd9vqai=hGuQ8kuc9pgc9s8qqaq=dirpe0xb9q8qiLsFr0=vr0=vr0dc8meaabaqaciaacaGaaeqabaqabeGadaaakeaadaqiaaqaaiabdsfaujabdcfaqbGaayPadaaaaa@2FC8@ = 203.67 since it is closest to the true number (200) of DE genes. In this case, mean FP_
 MathType@MTEF@5@5@+=feaafiart1ev1aaatCvAUfKttLearuWrP9MDH5MBPbIqV92AaeXatLxBI9gBaebbnrfifHhDYfgasaacH8akY=wiFfYdH8Gipec8Eeeu0xXdbba9frFj0=OqFfea0dXdd9vqai=hGuQ8kuc9pgc9s8qqaq=dirpe0xb9q8qiLsFr0=vr0=vr0dc8meaabaqaciaacaGaaeqabaqabeGadaaakeaadaqiaaqaaiabdAeagjabdcfaqbGaayPadaaaaa@2FAC@ from (7) is about 6 which is approximately 1/4 of the mean number of true FP.

**Table 1:** Estimated numbers of FP from SAM, (7) and sam2.20, *s*_0 _default choice of SAM. Table 1 displays the average numbers of TP_
 MathType@MTEF@5@5@+=feaafiart1ev1aaatCvAUfKttLearuWrP9MDH5MBPbIqV92AaeXatLxBI9gBaebbnrfifHhDYfgasaacH8akY=wiFfYdH8Gipec8Eeeu0xXdbba9frFj0=OqFfea0dXdd9vqai=hGuQ8kuc9pgc9s8qqaq=dirpe0xb9q8qiLsFr0=vr0=vr0dc8meaabaqaciaacaGaaeqabaqabeGadaaakeaadaqiaaqaaiabdsfaujabdcfaqbGaayPadaaaaa@2FC8@, true FP and the estimated FP from SAM, formula (7) and sam2.20 from 100 simulations at different levels of estimated TP.

Mean TP_ MathType@MTEF@5@5@+=feaafiart1ev1aaatCvAUfKttLearuWrP9MDH5MBPbIqV92AaeXatLxBI9gBaebbnrfifHhDYfgasaacH8akY=wiFfYdH8Gipec8Eeeu0xXdbba9frFj0=OqFfea0dXdd9vqai=hGuQ8kuc9pgc9s8qqaq=dirpe0xb9q8qiLsFr0=vr0=vr0dc8meaabaqaciaacaGaaeqabaqabeGadaaakeaadaqiaaqaaiabdsfaujabdcfaqbGaayPadaaaaa@2FC8@	Mean of true FP	Mean FP_ MathType@MTEF@5@5@+=feaafiart1ev1aaatCvAUfKttLearuWrP9MDH5MBPbIqV92AaeXatLxBI9gBaebbnrfifHhDYfgasaacH8akY=wiFfYdH8Gipec8Eeeu0xXdbba9frFj0=OqFfea0dXdd9vqai=hGuQ8kuc9pgc9s8qqaq=dirpe0xb9q8qiLsFr0=vr0=vr0dc8meaabaqaciaacaGaaeqabaqabeGadaaakeaadaqiaaqaaiabdAeagjabdcfaqbGaayPadaaaaa@2FAC@ from SAM	Mean FP_ MathType@MTEF@5@5@+=feaafiart1ev1aaatCvAUfKttLearuWrP9MDH5MBPbIqV92AaeXatLxBI9gBaebbnrfifHhDYfgasaacH8akY=wiFfYdH8Gipec8Eeeu0xXdbba9frFj0=OqFfea0dXdd9vqai=hGuQ8kuc9pgc9s8qqaq=dirpe0xb9q8qiLsFr0=vr0=vr0dc8meaabaqaciaacaGaaeqabaqabeGadaaakeaadaqiaaqaaiabdAeagjabdcfaqbGaayPadaaaaa@2FAC@ from (7)	Mean FP_ MathType@MTEF@5@5@+=feaafiart1ev1aaatCvAUfKttLearuWrP9MDH5MBPbIqV92AaeXatLxBI9gBaebbnrfifHhDYfgasaacH8akY=wiFfYdH8Gipec8Eeeu0xXdbba9frFj0=OqFfea0dXdd9vqai=hGuQ8kuc9pgc9s8qqaq=dirpe0xb9q8qiLsFr0=vr0=vr0dc8meaabaqaciaacaGaaeqabaqabeGadaaakeaadaqiaaqaaiabdAeagjabdcfaqbGaayPadaaaaa@2FAC@ from sam2.20
248.31	59.32	20.73	7.96	68.71
203.67	24.06	12.37	6.21	28.98
152.03	4.61	5.55	4.34	5.41

The above discussion shows that formula (7), which uses the displacement between the order null scores and the expected null scores to declare significance, does not generally provide meaningful estimates of the number of true FP. This is probably what motivated SAM to use (6), instead of (7), to declare significance of the null scores. Column 3 of Table 1 reports the FP estimates obtained by using (6). Unfortunately, it can be seen that the same under-estimation problem also exists for (6), although to a lesser degree. As explained in Subsection 2.3.2 below, the underestimation of (6) is caused by the use of only predicted DE genes in the estimation of FP. This issue has been resolved in sam2.20 by switching to (9) of Step (f'). The last column of Table 1 shows the results from sam2.20. The improvement made by sam2.20 is obvious despite the obvious over-estimation problem. Due to the obvious weakness of SAM, our future discussion will be focused on sam2.20.

### 3.2 The conflicting results of sam2.20 due to the use of asymmetric cutoffs

A re-visit of Figures 1 and 2 reveals that sam2.20 may produce conflicting results. For example, the point (see the red point outside the interval d¯(1)
 MathType@MTEF@5@5@+=feaafiart1ev1aaatCvAUfKttLearuWrP9MDH5MBPbIqV92AaeXatLxBI9gBaebbnrfifHhDYfgasaacH8akY=wiFfYdH8Gipec8Eeeu0xXdbba9frFj0=OqFfea0dXdd9vqai=hGuQ8kuc9pgc9s8qqaq=dirpe0xb9q8qiLsFr0=vr0=vr0dc8meaabaqaciaacaGaaeqabaqabeGadaaakeaacuWGKbazgaqeamaaBaaaleaacqGGOaakcqaIXaqmcqGGPaqkaeqaaaaa@30E3@ ± Δ at the lower left corner of Figure 1)) is not considered significant negative (see Figure 2) by sam2.20 since the point is above, instead of below, the band d¯(i)
 MathType@MTEF@5@5@+=feaafiart1ev1aaatCvAUfKttLearuWrP9MDH5MBPbIqV92AaeXatLxBI9gBaebbnrfifHhDYfgasaacH8akY=wiFfYdH8Gipec8Eeeu0xXdbba9frFj0=OqFfea0dXdd9vqai=hGuQ8kuc9pgc9s8qqaq=dirpe0xb9q8qiLsFr0=vr0=vr0dc8meaabaqaciaacaGaaeqabaqabeGadaaakeaacuWGKbazgaqeamaaBaaaleaacqGGOaakcqWGPbqAcqGGPaqkaeqaaaaa@314E@ ± Δ despite the fact that it has displacement larger than Δ. If the same logic applies, the last 2 points (see the red points on the upper right corner below d¯(i)
 MathType@MTEF@5@5@+=feaafiart1ev1aaatCvAUfKttLearuWrP9MDH5MBPbIqV92AaeXatLxBI9gBaebbnrfifHhDYfgasaacH8akY=wiFfYdH8Gipec8Eeeu0xXdbba9frFj0=OqFfea0dXdd9vqai=hGuQ8kuc9pgc9s8qqaq=dirpe0xb9q8qiLsFr0=vr0=vr0dc8meaabaqaciaacaGaaeqabaqabeGadaaakeaacuWGKbazgaqeamaaBaaaleaacqGGOaakcqWGPbqAcqGGPaqkaeqaaaaa@314E@ - Δ, Figure 1) should not be considered significant positive, either. However, sam2.20 declared these two including all the other 21 points within the band d¯(i)
 MathType@MTEF@5@5@+=feaafiart1ev1aaatCvAUfKttLearuWrP9MDH5MBPbIqV92AaeXatLxBI9gBaebbnrfifHhDYfgasaacH8akY=wiFfYdH8Gipec8Eeeu0xXdbba9frFj0=OqFfea0dXdd9vqai=hGuQ8kuc9pgc9s8qqaq=dirpe0xb9q8qiLsFr0=vr0=vr0dc8meaabaqaciaacaGaaeqabaqabeGadaaakeaacuWGKbazgaqeamaaBaaaleaacqGGOaakcqWGPbqAcqGGPaqkaeqaaaaa@314E@ ± Δ significant since they are larger than the upper cutoff *δ*_*U *_= 1.3068 (Figure 2). Such contradiction makes the interpretation of the results very difficult. As a matter of fact, if one uses a slightly higher threshold Δ, all these 24 points above *δ*_*U *_= 1.3068 will be declared non-significant, hence, causes a sudden big drop in the number of significant genes. Such phenomenon is often seen in the output of sam2.20.

Ideally, one would expect that the ordered test statistics stay above or below the band d¯(i)
 MathType@MTEF@5@5@+=feaafiart1ev1aaatCvAUfKttLearuWrP9MDH5MBPbIqV92AaeXatLxBI9gBaebbnrfifHhDYfgasaacH8akY=wiFfYdH8Gipec8Eeeu0xXdbba9frFj0=OqFfea0dXdd9vqai=hGuQ8kuc9pgc9s8qqaq=dirpe0xb9q8qiLsFr0=vr0=vr0dc8meaabaqaciaacaGaaeqabaqabeGadaaakeaacuWGKbazgaqeamaaBaaaleaacqGGOaakcqWGPbqAcqGGPaqkaeqaaaaa@314E@ ± Δ once they cross it. However, Figures 1 and 2 show that such an ideal scenario may not always happen. This un-predictability of the ordered test statistics is caused by the larger variability of the ordered test statistics *d*_(*i*) _for *i *near 1 or *n*. Denote the probability density function and the cumulative distribution function of a random variable *d *by *f *and *F*, respectively. Then, for the *i*th order statistic *d*_(*i*)_, it can be shown [23] that

E[d(i)]≈F−1(iN+1) and Var[d(i)]≈iN+1(1−iN+1)(1N+2)/(f[F−1(iN+1)])2.
 MathType@MTEF@5@5@+=feaafiart1ev1aaatCvAUfKttLearuWrP9MDH5MBPbIqV92AaeXatLxBI9gBaebbnrfifHhDYfgasaacH8akY=wiFfYdH8Gipec8Eeeu0xXdbba9frFj0=OqFfea0dXdd9vqai=hGuQ8kuc9pgc9s8qqaq=dirpe0xb9q8qiLsFr0=vr0=vr0dc8meaabaqaciaacaGaaeqabaqabeGadaaakeaacqWGfbqrcqGGBbWwcqWGKbazdaWgaaWcbaGaeiikaGIaemyAaKMaeiykaKcabeaakiabc2faDjabgIKi7kabdAeagnaaCaaaleqabaGaeyOeI0IaeGymaedaaOGaeiikaGYaaSaaaeaacqWGPbqAaeaacqWGobGtcqGHRaWkcqaIXaqmaaGaeiykaKIaeeiiaaIaeeyyaeMaeeOBa4MaeeizaqMaeeiiaaIaeeOvayLaeeyyaeMaeeOCaiNaei4waSLaemizaq2aaSbaaSqaaiabcIcaOiabdMgaPjabcMcaPaqabaGccqGGDbqxcqGHijYUdaWcaaqaaiabdMgaPbqaaiabd6eaojabgUcaRiabigdaXaaacqGGOaakcqaIXaqmcqGHsisldaWcaaqaaiabdMgaPbqaaiabd6eaojabgUcaRiabigdaXaaacqGGPaqkcqGGOaakdaWcaaqaaiabigdaXaqaaiabd6eaojabgUcaRiabikdaYaaacqGGPaqkcqGGVaWldaqadaqaaiabdAgaMnaadmaabaGaemOray0aaWbaaSqabeaacqGHsislcqaIXaqmaaGccqGGOaakdaWcaaqaaiabdMgaPbqaaiabd6eaojabgUcaRiabigdaXaaacqGGPaqkaiaawUfacaGLDbaaaiaawIcacaGLPaaadaahaaWcbeqaaiabikdaYaaakiabc6caUaaa@74F4@

**Figure 3:**
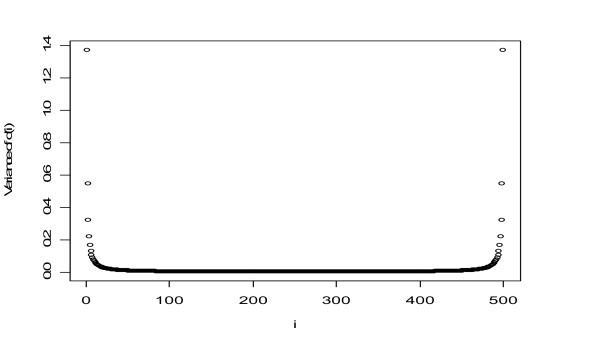
**Plot of the variance of the ordered test statistics *d*_(*i*)_**. The vertical axis displays the values of the variances of the order statistics *d*_(*i*) _for *i *= 1, ..., 500.

Figure 3 shows the values of Var[*d*_(*i*)_] (*i *= 1, ..., 500) for 500 observations from the *t*-distribution with 5 degrees of freedom. It can be seen that the variability of the ordered statistics at both ends are significantly larger than that of the ordered statistics in the middle.

The same variability was also observed in [12] by numerically comparing *d*_(*i*) _with the true expected order statistics. The significantly larger variability of the order statistic *d*_(*i*) _for *i *near 1 and *n *may cause such genes to have a higher probability of being falsely claimed significant. Since the threshold Δ is not directly applied on the test statistics, another side-effect of using the displacement of the ordered test statistics from the expected null scores is that sam2.20 cannot provide FDR estimates for consecutive values of TP_
 MathType@MTEF@5@5@+=feaafiart1ev1aaatCvAUfKttLearuWrP9MDH5MBPbIqV92AaeXatLxBI9gBaebbnrfifHhDYfgasaacH8akY=wiFfYdH8Gipec8Eeeu0xXdbba9frFj0=OqFfea0dXdd9vqai=hGuQ8kuc9pgc9s8qqaq=dirpe0xb9q8qiLsFr0=vr0=vr0dc8meaabaqaciaacaGaaeqabaqabeGadaaakeaadaqiaaqaaiabdsfaujabdcfaqbGaayPadaaaaa@2FC8@. This is related to the previous discussion on the possible sudden drop in the number of significant genes which sam2.20 declares.

Note that the only function of using the displacement of the ordered test statistics from the expected null scores in sam2.20 is to obtain the cutoffs. Such cutoffs are often asymmetric. It was claimed in [9] that the asymmetric cutoffs have the advantage over the conventional symmetric cutoff method. The reason is that the induced and repressed genes may behave differently in some biological experiments. To verify this claim, we carried out simulations in which different probability distributions were used for the induced and repressed genes (Setups 2–3 of Table 4). The simulation results show that the asymmetric cutoffs do have advantage over the symmetric cutoff method under Setups 2 (i) and 3(i). However, the advantage in this case is not caused by the asymmetric distributions of the induced and repressed genes, but by the fact that the DE genes are either all induced or all repressed genes under these two setups. For a dataset with equal number of symmetrically distributed induced and repressed genes, the asymmetric cutoffs may result in significantly asymmetric numbers of significant positive and negative genes. Table 2 illustrates such a scenario. The data used in producing Table 2 were generated according to model (10) under the same setup as that used in obtaining Figures 1 and 2, in which 100 induced and 100 repressed genes were symmetrically generated on the positive and negative sides. A total of 100 simulations were carried out to examine the numbers of significant positive and negative genes identified by sam2.20 given the threshold Δ = 0.035. The total numbers of significant genes at this threshold ranges from 17 to 854 with mean 207.67. In each simulation, we counted the number of significant positive and negative genes. The mean numbers of significant positive and negative genes from 100 simulations are 106.77 and 100.9, respectively. This difference does not show strong evidence of asymmetry in the numbers of significant positive and negative genes. However, checking the numbers of significant positive and negative genes from each simulation reveals that sam2.20 can be very unpredictable in identifying significant positive and negative genes. Take Column 7 (Simulation 6) for example, sam2.20 reported 380 significant positive and only 14 significant negative genes even when the number of total significant genes is as large as 394.

**Table 2:** Numbers of significant positive and negative genes identified by sam2.20 at Δ = 0.035. Table 2 displays the numbers of significant positive and negative genes from 10 simulations under the same setup as that used in producing Figures 1 and 2.

Simulation	1	2	3	4	5	6	7	8	9	10
Number of sig. genes	230	158	294	75	468	394	41	168	74	206
sig. pos	73	27	140	75	285	380	34	86	26	129
sig. neg	157	131	154	0	183	14	7	82	48	77

**Table 4:** Results obtained under Setups (1) – (3). The medians reported in the table were calculated from 100 simulations. The median est.FDR values reported in Column 4 were obtained from sam2.20 and the symmetric cutoff method directly. The median est.FDRc values reported in Column 5 are the estimated FDR with FP correction (12).

Setup		Median *M*	Median true FDR sam2.20/sym.cutoff	Median est.FDR sam2.20/sym.cut	Median est. FDRc sam2.20/sym.cut
1		199	0.1005/0.1005	0.1188/0.1238	0.0981/0.1020
2	(i)	210.5	0.7082/0.7779	0.6959/0.8130	0.6875/0.7945
	(ii)	211.5	0.6699/0.6748	0.6730/0.7219	0.6331/0.6933
	(iii)	207	0.6578/0.6533	0.6738/0.7039	0.6376/0.6616
	(iv)	200	0.5025/0.4371	0.5305/0.4846	0.5004/0.4403
3	(i)	399	0.0599/0.0800	0.0596/0.1017	0.0598/0.0779
	(ii)	399	0.1827/0.1754	0.1874/0.2135	0.1862/0.1758
	(iii)	399	0.2130/0.1989	0.21750/.2462	0.2168/0.2007
	(iv)	400.5	0.4913/0.3950	0.5067/0.4511	0.4975/0.3994

### 3.3 The complications caused by the use of the same SAM statistic as both the test and null statistics

An immediate effect of the use of the same SAM statistic as both the test and null statistic is the over-dispersed null scores. In the two experimental condition comparisons, a simple permutation is applied to the *K *+ *J *expression levels of a gene, and the first *K *expression levels will be treated as the observations under experimental condition 1 and the last *J *expression levels as the observations under experimental condition 2. Then, the SAM statistic is applied to the permuted data to obtain the null scores.

By definition, a null distribution should be irrelevant of the experimental conditions. Hence, the null distribution under model (10) should have mean zero regardless of the experimental conditions. Note that the first *K *and last *J *expression levels in the permuted data are usually the mixtures of expression levels under both experimental conditions, respectively. The set of null scores generated from such permutation may have a non-zero mean, hence leading to over-dispersed null scores. In some cases, it was suggested to only use the sets of null scores generated from certain appropriate permutations. For example, the concept of balanced permutation was proposed in [9,11] to insure the null scores generated to have mean 0. However, this suggestion was only specific to the ionizing radiation response experiment considered in those papers. No general rules were provided.

Since the original ordering {1, ..., *J*; (*J *+ 1), ..., (*J *+ *K*)} is one of the permutations used in sam2.20, the use of such permutation and the use of the same SAM statistic as both the test and null statistic would count an estimated TP case as an FP case in the estimation of FDR. Actually TP_
 MathType@MTEF@5@5@+=feaafiart1ev1aaatCvAUfKttLearuWrP9MDH5MBPbIqV92AaeXatLxBI9gBaebbnrfifHhDYfgasaacH8akY=wiFfYdH8Gipec8Eeeu0xXdbba9frFj0=OqFfea0dXdd9vqai=hGuQ8kuc9pgc9s8qqaq=dirpe0xb9q8qiLsFr0=vr0=vr0dc8meaabaqaciaacaGaaeqabaqabeGadaaakeaadaqiaaqaaiabdsfaujabdcfaqbGaayPadaaaaa@2FC8@ is the maximum value of the FP_
 MathType@MTEF@5@5@+=feaafiart1ev1aaatCvAUfKttLearuWrP9MDH5MBPbIqV92AaeXatLxBI9gBaebbnrfifHhDYfgasaacH8akY=wiFfYdH8Gipec8Eeeu0xXdbba9frFj0=OqFfea0dXdd9vqai=hGuQ8kuc9pgc9s8qqaq=dirpe0xb9q8qiLsFr0=vr0=vr0dc8meaabaqaciaacaGaaeqabaqabeGadaaakeaadaqiaaqaaiabdAeagjabdcfaqbGaayPadaaaaa@2FAC@ values whose median is used in the estimation of FDR_
 MathType@MTEF@5@5@+=feaafiart1ev1aaatCvAUfKttLearuWrP9MDH5MBPbIqV92AaeXatLxBI9gBaebbnrfifHhDYfgasaacH8akY=wiFfYdH8Gipec8Eeeu0xXdbba9frFj0=OqFfea0dXdd9vqai=hGuQ8kuc9pgc9s8qqaq=dirpe0xb9q8qiLsFr0=vr0=vr0dc8meaabaqaciaacaGaaeqabaqabeGadaaakeaadaqiaaqaaiabdAeagjabdseaejabdkfasbGaayPadaaaaa@30C1@. A permutation not too different from {1, ..., *J*; (*J *+ 1), ..., (*J *+ *K*)} would give FP_
 MathType@MTEF@5@5@+=feaafiart1ev1aaatCvAUfKttLearuWrP9MDH5MBPbIqV92AaeXatLxBI9gBaebbnrfifHhDYfgasaacH8akY=wiFfYdH8Gipec8Eeeu0xXdbba9frFj0=OqFfea0dXdd9vqai=hGuQ8kuc9pgc9s8qqaq=dirpe0xb9q8qiLsFr0=vr0=vr0dc8meaabaqaciaacaGaaeqabaqabeGadaaakeaadaqiaaqaaiabdAeagjabdcfaqbGaayPadaaaaa@2FAC@ similar to TP_
 MathType@MTEF@5@5@+=feaafiart1ev1aaatCvAUfKttLearuWrP9MDH5MBPbIqV92AaeXatLxBI9gBaebbnrfifHhDYfgasaacH8akY=wiFfYdH8Gipec8Eeeu0xXdbba9frFj0=OqFfea0dXdd9vqai=hGuQ8kuc9pgc9s8qqaq=dirpe0xb9q8qiLsFr0=vr0=vr0dc8meaabaqaciaacaGaaeqabaqabeGadaaakeaadaqiaaqaaiabdsfaujabdcfaqbGaayPadaaaaa@2FC8@. This shows that the 90th FP_
 MathType@MTEF@5@5@+=feaafiart1ev1aaatCvAUfKttLearuWrP9MDH5MBPbIqV92AaeXatLxBI9gBaebbnrfifHhDYfgasaacH8akY=wiFfYdH8Gipec8Eeeu0xXdbba9frFj0=OqFfea0dXdd9vqai=hGuQ8kuc9pgc9s8qqaq=dirpe0xb9q8qiLsFr0=vr0=vr0dc8meaabaqaciaacaGaaeqabaqabeGadaaakeaadaqiaaqaaiabdAeagjabdcfaqbGaayPadaaaaa@2FAC@ reported in sam2.20 would significantly over-estimate the true 90th FP and is not much informative. This also explains why sam2.20 uses the median FDR_
 MathType@MTEF@5@5@+=feaafiart1ev1aaatCvAUfKttLearuWrP9MDH5MBPbIqV92AaeXatLxBI9gBaebbnrfifHhDYfgasaacH8akY=wiFfYdH8Gipec8Eeeu0xXdbba9frFj0=OqFfea0dXdd9vqai=hGuQ8kuc9pgc9s8qqaq=dirpe0xb9q8qiLsFr0=vr0=vr0dc8meaabaqaciaacaGaaeqabaqabeGadaaakeaadaqiaaqaaiabdAeagjabdseaejabdkfasbGaayPadaaaaa@30C1@ (instead of the mean) of all the FDR_
 MathType@MTEF@5@5@+=feaafiart1ev1aaatCvAUfKttLearuWrP9MDH5MBPbIqV92AaeXatLxBI9gBaebbnrfifHhDYfgasaacH8akY=wiFfYdH8Gipec8Eeeu0xXdbba9frFj0=OqFfea0dXdd9vqai=hGuQ8kuc9pgc9s8qqaq=dirpe0xb9q8qiLsFr0=vr0=vr0dc8meaabaqaciaacaGaaeqabaqabeGadaaakeaadaqiaaqaaiabdAeagjabdseaejabdkfasbGaayPadaaaaa@30C1@ values from all the permutations as the estimate of FDR. The use of the mean FDR_
 MathType@MTEF@5@5@+=feaafiart1ev1aaatCvAUfKttLearuWrP9MDH5MBPbIqV92AaeXatLxBI9gBaebbnrfifHhDYfgasaacH8akY=wiFfYdH8Gipec8Eeeu0xXdbba9frFj0=OqFfea0dXdd9vqai=hGuQ8kuc9pgc9s8qqaq=dirpe0xb9q8qiLsFr0=vr0=vr0dc8meaabaqaciaacaGaaeqabaqabeGadaaakeaadaqiaaqaaiabdAeagjabdseaejabdkfasbGaayPadaaaaa@30C1@ value would lead to a much inflated estimate of FDR.

**Figure 5:**
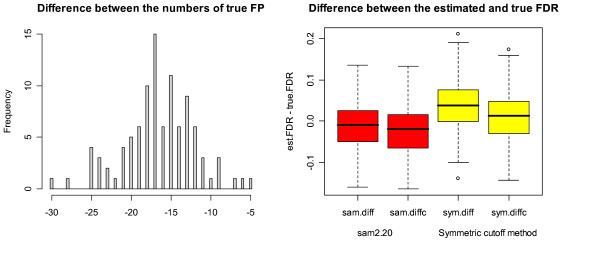
**Comparison between sam2.20 and the symmetric cutoff method under Setup 2(i)**. The histogram and the parallel boxplots in Figure 5 are defined the same as in Figure 4. The histogram shows that sam2.20 produces significantly smaller number of true FP than the symmetric cutoff method among all 100 simulations. The first two boxplots at the right of Figure 5 show that sam2.20 under-estimates the true FDR and FP correction (12) made the under-estimation even worse. The last two boxplots at the right of Figure 5 show that the symmetric cutoff method over-estimates the true FDR and the over-estimation has been corrected by FP correction (12).

The use of the same SAM statistic as both the test and null statistic has another complication. Note that the numerator and the denominator of the SAM statistic are independent under the assumption that the data obtained in the microarray experiment are normally distributed. However, such independence is lost when the SAM statistic is applied to the permuted data to generate the null scores. The loss of the independence may cause under-estimation of FDR (Figure 5).

## 4 The symmetric cutoff method

The discussion in Section 3 shows that the contradictory results of sam2.20 can be avoided without the use of the order statistics in searching for the cutoffs. Note that the theoretical null distribution is symmetric about 0 under the mild assumption that the errors in model (10) are symmetric about 0. This means that the use of symmetric cutoffs in declaring significance actually makes more sense than the use of asymmetric cutoffs. This motivates us to re-visit the conventional symmetric cutoff method. For simplicity, we shall call it the symmetric cutoff method. For clarity, we describe the algorithm of the symmetric cutoff method below.

In practice, it is quite common that scientists have certain knowledge about a reasonable range of the number of differentially expressed genes. Denote the minimum and maximum numbers of significant genes the scientist would like to consider by *M*_0 _and *M*_1_, respectively. Assume that we have obtained *s*_0 _as in sam2.20, the symmetric cutoff method uses the following algorithm:

1. Calculate the test statistics *d*(1), ..., *d*(*n*) from (1) for all genes.

2. Given any TP_
 MathType@MTEF@5@5@+=feaafiart1ev1aaatCvAUfKttLearuWrP9MDH5MBPbIqV92AaeXatLxBI9gBaebbnrfifHhDYfgasaacH8akY=wiFfYdH8Gipec8Eeeu0xXdbba9frFj0=OqFfea0dXdd9vqai=hGuQ8kuc9pgc9s8qqaq=dirpe0xb9q8qiLsFr0=vr0=vr0dc8meaabaqaciaacaGaaeqabaqabeGadaaakeaadaqiaaqaaiabdsfaujabdcfaqbGaayPadaaaaa@2FC8@ = *M *∈ [*M*_0_, *M*_1_], declare the *M *genes with the largest test statistics in their absolute values as the significant genes.

3. Define the symmetric cutoffs *δ*_*U *_= -*δ*_*L *_= *ν*, where *ν *is the smallest value among the absolute values of the test statistics from the genes declared significant.

4. The FDR is estimated by FDR_=π^0FP_
 MathType@MTEF@5@5@+=feaafiart1ev1aaatCvAUfKttLearuWrP9MDH5MBPbIqV92AaeXatLxBI9gBaebbnrfifHhDYfgasaacH8akY=wiFfYdH8Gipec8Eeeu0xXdbba9frFj0=OqFfea0dXdd9vqai=hGuQ8kuc9pgc9s8qqaq=dirpe0xb9q8qiLsFr0=vr0=vr0dc8meaabaqaciaacaGaaeqabaqabeGadaaakeaadaqiaaqaaiabdAeagjabdseaejabdkfasbGaayPadaGaeyypa0dcciGaf8hWdaNbaKaadaWgaaWcbaGaeGimaadabeaakmaaHaaabaGaemOrayKaemiuaafacaGLcmaaaaa@37BF@/*M*, with

FP_=median(FP_(1),⋯,FP_(B)),
 MathType@MTEF@5@5@+=feaafiart1ev1aaatCvAUfKttLearuWrP9MDH5MBPbIqV92AaeXatLxBI9gBaebbnrfifHhDYfgasaacH8akY=wiFfYdH8Gipec8Eeeu0xXdbba9frFj0=OqFfea0dXdd9vqai=hGuQ8kuc9pgc9s8qqaq=dirpe0xb9q8qiLsFr0=vr0=vr0dc8meaabaqaciaacaGaaeqabaqabeGadaaakeaadaqiaaqaaiabdAeagjabdcfaqbGaayPadaGaeyypa0JaeeyBa0MaeeyzauMaeeizaqMaeeyAaKMaeeyyaeMaeeOBa42aaeWaaeaadaqiaaqaaiabdAeagjabdcfaqbGaayPadaGaeiikaGIaeGymaeJaeiykaKIaeiilaWIaeS47IWKaeiilaWYaaecaaeaacqWGgbGrcqWGqbauaiaawkWaaiabcIcaOiabdkeacjabcMcaPaGaayjkaiaawMcaaiabcYcaSaaa@4A30@

where FP_
 MathType@MTEF@5@5@+=feaafiart1ev1aaatCvAUfKttLearuWrP9MDH5MBPbIqV92AaeXatLxBI9gBaebbnrfifHhDYfgasaacH8akY=wiFfYdH8Gipec8Eeeu0xXdbba9frFj0=OqFfea0dXdd9vqai=hGuQ8kuc9pgc9s8qqaq=dirpe0xb9q8qiLsFr0=vr0=vr0dc8meaabaqaciaacaGaaeqabaqabeGadaaakeaadaqiaaqaaiabdAeagjabdcfaqbGaayPadaaaaa@2FAC@(*b*) = #{1 ≤ *i *≤ *n*: *d*^*b*^(*i*) > *δ*_*U *_or *d*^*b*^(*i*) <*δ*_*L*_} (*b *= 1, ..., *B*) and *d*^*b*^(*i*), *i *= 1, ..., *n*, are the null scores calculated from (3) corresponding to permutation *b *and *B *is the total number of permutations used.

In the above algorithm, we still use the same SAM statistic and the same simple permutations as those used in sam2.20. However, as discussed before, the use of the same SAM statistic and the use of simple permutations may cause over-estimation of FP. To correct the over-estimation problem, following the idea of [14], we propose the following formula to estimate FDR:

5. The FDR is estimated by FDR_=π^0FP_
 MathType@MTEF@5@5@+=feaafiart1ev1aaatCvAUfKttLearuWrP9MDH5MBPbIqV92AaeXatLxBI9gBaebbnrfifHhDYfgasaacH8akY=wiFfYdH8Gipec8Eeeu0xXdbba9frFj0=OqFfea0dXdd9vqai=hGuQ8kuc9pgc9s8qqaq=dirpe0xb9q8qiLsFr0=vr0=vr0dc8meaabaqaciaacaGaaeqabaqabeGadaaakeaadaqiaaqaaiabdAeagjabdseaejabdkfasbGaayPadaGaeyypa0dcciGaf8hWdaNbaKaadaWgaaWcbaGaeGimaadabeaakmaaHaaabaGaemOrayKaemiuaafacaGLcmaaaaa@37BF@/*M*, where

FP_=n#of genes in T′median(FP′_(1),⋯,FP′_(B)),
 MathType@MTEF@5@5@+=feaafiart1ev1aaatCvAUfKttLearuWrP9MDH5MBPbIqV92AaeXatLxBI9gBaebbnrfifHhDYfgasaacH8akY=wiFfYdH8Gipec8Eeeu0xXdbba9frFj0=OqFfea0dXdd9vqai=hGuQ8kuc9pgc9s8qqaq=dirpe0xb9q8qiLsFr0=vr0=vr0dc8meaabaqaciaacaGaaeqabaqabeGadaaakeaadaqiaaqaaiabdAeagjabdcfaqbGaayPadaGaeyypa0ZaaSaaaeaacqWGUbGBaeaacqGGJaWicqqGVbWBcqqGMbGzcqqGGaaicqqGNbWzcqqGLbqzcqqGUbGBcqqGLbqzcqqGZbWCcqqGGaaicqqGPbqAcqqGUbGBcqqGGaaicuWGubavgaqbaaaacqqGTbqBcqqGLbqzcqqGKbazcqqGPbqAcqqGHbqycqqGUbGBdaqadaqaamaaHaaabaGaemOrayKafmiuaaLbauaaaiaawkWaaiabcIcaOiabigdaXiabcMcaPiabcYcaSiabl+UimjabcYcaSmaaHaaabaGaemOrayKafmiuaaLbauaaaiaawkWaaiabcIcaOiabdkeacjabcMcaPaGaayjkaiaawMcaaiabcYcaSaaa@5C58@

where FP′_
 MathType@MTEF@5@5@+=feaafiart1ev1aaatCvAUfKttLearuWrP9MDH5MBPbIqV92AaeXatLxBI9gBaebbnrfifHhDYfgasaacH8akY=wiFfYdH8Gipec8Eeeu0xXdbba9frFj0=OqFfea0dXdd9vqai=hGuQ8kuc9pgc9s8qqaq=dirpe0xb9q8qiLsFr0=vr0=vr0dc8meaabaqaciaacaGaaeqabaqabeGadaaakeaadaqiaaqaaiabdAeagjqbdcfaqzaafaaacaGLcmaaaaa@2FB8@(*b*)= #{*i *∈ *T*': *d*^*b*^(*i*) > *δ*_*U *_or *d*^*b*^(*i*) <*δ*_*L*_} (*b *= 1, ..., *B*) and the set *T*' in (12) is the set of all genes after removing the genes declared significant in Step (2).

The symmetric cutoff method with the use of (12) will be called the symmetric cutoff method with FP correction. For comparison, we also applied (12) to the results from sam2.20.

## 5 Numerical results

In this section, we provide a comparison between sam2.20 and the symmetric cutoff method under different setups. Our numerical comparisons are organized as follows. Under each setup, we generate *n *(= 5000) genes from model (10) with *J *= 4 and *K *= 4. Then, we calculate the true FDR, the estimated FDR and the estimated FDR with FP correction, given a specific number (*M*) of significant genes. It is obvious that the performance of FP correction (12) depends on the magnitude of *M*. If *M *is much larger than the number of true DE genes in the microarray data, the exclusion of the *M *genes will cause significant under-estimation of the true number of FP (and FDR). On the other hand, if *M *is much smaller than the true number of DE genes, FP correction (12) will have little effect on correcting the over-estimation problem. However, it is reasonable to expect a proposed FP correction to perform well when *M *is close to the number of true DE genes. Due to this consideration, we decided to choose *M *as the number of the true DE genes in the simulations. Since it is not always possible for sam2.20 to find the threshold Δ that gives exactly *M *significant genes, the actual values of *M *realized may vary from simulation to simulation. This process is repeated 100 times. Table 3 provides a summary of the setups used in the simulations.

**Table 3:** Simulation setups. Under each setup, there are 5000 genes. Table 3 shows how the genes were simulated. For example, under setup 1, the first 100 genes were generated from *N*(0,1) and *N*(3,1) under experimental conditions 1 and 2, respectively, the middle 4800 genes were generated from *N*(0,1) regardless of experimental condition and the last 100 genes were generated from *N*(0,1) and *N*(-3,1) under experimental conditions 1 and 2, respectively. The third column displays the ratio of induced to repressed genes. If the number of repressed genes is 0, the ratio is defined as ∞.

Setup		Genes First/middle/last	Ratio	Experimental condition 1	Experimental condition 2
1		100/4800/100	1/1	*N*(0,1)/*N*(0,1)/*N*(0,1)	*N*(3,1)/*N*(0,1)/*N*(-3,1)
2	(i)	200/4800/0	∞		
	(ii)	167/4800/33	5/1		
	(iii)	160/4800/40	4/1		
	(iv)	100/4800/100	1/1	*N*(0,1)/*N*(0,1)/*N*(0,1)	*N*(1,1)/*N*(0,1)/*N*(-3,1)
3	(i)	0/4600/400	0		
	(ii)	66/4600/334	1/5		
	(iii)	80/4600/320	1/4		
	(iv)	200/4600/200	1/1		

**Figure 4:**
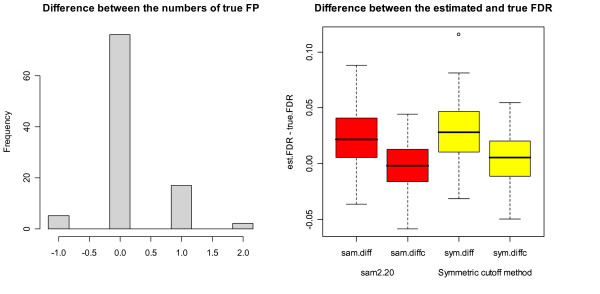
**Comparison between sam2.20 and the symmetric cutoff method under Setup 1**. The histogram at the left of Figure 4 shows the number of true FP from sam2.20 subtracted by the number of true FP from the symmetric cutoff method. The parallel boxplots at the right of Figure 4 are the boxplots of the values of 1) est. FDR from sam2.20 – the true FDR (sam.diff), 2) est. FDR from sam2.20 with FP correction – the true FDR (sam.diffc), 3) est. FDR from the symmetric cutoff method – true FDR (sym.diff) and 4) est. FDR from the symmetric cutoff method with FP correction – true FDR (sym.diffc).

**Figure 6:**
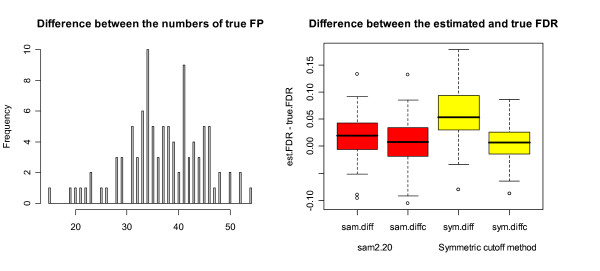
**Comparison between sam2.20 and the symmetric cutoff method under setup 3(iv)**. The histogram and the parallel boxplots in Figure 6 are defined the same as in Figure 4. It is clear from the histogram that sam2.20 tends to produce significantly higher number of true FP than the symmetric cutoff method. The parallel boxplots show that both methods over-estimate the true FDR. It can also been seen that the over-estimation for the symmetric cutoff method is more serious than that of sam2.20. The second and fourth boxplots at the right of Figure 6 show that the over-estimation has been corrected by FP correction (12) for both methods.

The results obtained from the simulations are summarized in Table 4. To be consistent with the results reported in the boxplots of Figures 4, 5, 6, we reported the medians instead of means in Table 4. It can be seen that the performance of the symmetric cutoff method and sam2.20 is very similar under Setup 1 with the same true FDR until the fourth digit after the decimal point. It can also be seen that both sam2.20 and the symmetric cutoff method over-estimate the FDR significantly. The difference between the performances of sam2.20 and the symmetric cutoff method and the effect of FP correction (12) can be seen more clearly in Figure 4. The histogram at the left of Figure 4 shows that, among 100 simulations, the two methods have exactly the same number of true FP for 76 times and sam2.20 has higher numbers of true FP for 19 times (one more FP for 17 times, 2 more FP twice) while the symmetric cutoff method has one more FP for 5 times. This shows that the symmetric cutoff method in general provides smaller number of true FP than sam2.20. The boxplots at the right of Figure 4 demonstrate the power of FP correction (12). For both methods, the correction has successfully corrected the over-estimation problem.

Table 4 also shows that sam2.20 works better only if the ratio of induced to repressed genes is far from 1:1 (see the results under Setups 2(i, ii) and 3(i)). Otherwise the symmetric cutoff method has advantage over sam2.20. Notice that the advantage of the symmetric cutoff method over sam2.20 under Setup 1 is not as obvious as that observed under Setups 2(iv) and 3(iv). This means that the relative performance of the symmetric cutoff method to sam2.20 is dependent on the distributions of induced and repressed genes. On the other hand, the results under Setups 2(ii) and 3(ii) show that the relative performance of the symmetric cutoff method to sam2.20 is also affected by the ratio of DE to EE genes.

To get more insight into how sam2.20 and the symmetric cutoff method perform differently under different setups, we obtained Figures 5 and 6 under Setups 2(i) and 3(iv). Figure 5 shows that the symmetric cutoff method can have as many as 30 more FP than that from sam2.20 among 200 genes called significant. On the other hand, Figure 6 shows that sam2.20 can have as many as 54 more FP than the symmetric cutoff method among 400 significant genes under Setup 3(iv). The boxplots at the right of Figure 5 show that sam2.20 actually under-estimates the true FDR slightly and FP correction (12) has caused more severe under-estimation in this case. We have not observed any under-estimation problem for the symmetric cutoff method in the simulations we carried out. It can be seen from Figure 5 that, although it provides smaller median true FDR than sam2.20, the un-corrected symmetric cutoff method has a much more serious over-estimation problem than sam2.20. Nevertheless, the over-estimation problem has been largely corrected by FP correction (12).

Finally, we applied sam2.20 and the symmetric cutoff method to the well-known leukaemia data of Golub et al. [21], which studied the classification of acute leukaemias into those arising from lymphoid precursors (acute lymphoblastic leukaemia, ALL) or from myeloid precursors (acute myeloid leukaemia, AML). We used all 27 of ALL and 11 of AML samples in our calculation. For each sample (or array), the original data were pre-processed by first subtracting its median and then this difference was divided by its quartile range (the difference between the first and third quartiles). The results from both methods are reported in Table 5.

**Table 5:** Results obtained for the leukaemia data. Table 5 reports the number of significant positive and negative genes (columns 2, 6), the cutoffs (columns 3, 7), the estimated FDR (FDR) and the estimated FDR with FP correction (FDR-c) from sam2.20 and the symmetric cutoff method (columns 4, 8). Column 5 reports the number of genes found significant by sam2.20 and the symmetric cutoff method from the list of informative genes [21].

TP_ MathType@MTEF@5@5@+=feaafiart1ev1aaatCvAUfKttLearuWrP9MDH5MBPbIqV92AaeXatLxBI9gBaebbnrfifHhDYfgasaacH8akY=wiFfYdH8Gipec8Eeeu0xXdbba9frFj0=OqFfea0dXdd9vqai=hGuQ8kuc9pgc9s8qqaq=dirpe0xb9q8qiLsFr0=vr0=vr0dc8meaabaqaciaacaGaaeqabaqabeGadaaakeaadaqiaaqaaiabdsfaujabdcfaqbGaayPadaaaaa@2FC8@	sam2.20			# of genes from Golub et al.'s list	The symmetric cutoff method		
	
	sig. pos	cutup	FDR	sam2.20	sig. pos	cutup	FDR
	sig. neg	cutlo	FDR-c	sym.cut	sig. neg	cutlo	FDR-c
316	227	3.2023	0.0065	14	215	3.3073	0.0043
	89	-3.3888	0.0068	14	101	-3.3073	0.0045
191	154	3.8648	0.0036	9	143	3.9612	0.0036
	37	-4.2450	0.0037	11	48	-3.9612	0.0037
92	87	4.0787	0	7	73	4.1906	0
	5	-5.1595	0	9	19	-4.1906	0
29	29	5.3143	0	5	27	5.3685	0
	0	-*Inf*	0	6	2	-5.3685	0
23	23	5.5514	0	5	22	5.5678	0
	0	-*Inf*	0	5	1	-5.5678	0

Table 5 shows that the performance of the two methods is quite similar, except for the case when TP_
 MathType@MTEF@5@5@+=feaafiart1ev1aaatCvAUfKttLearuWrP9MDH5MBPbIqV92AaeXatLxBI9gBaebbnrfifHhDYfgasaacH8akY=wiFfYdH8Gipec8Eeeu0xXdbba9frFj0=OqFfea0dXdd9vqai=hGuQ8kuc9pgc9s8qqaq=dirpe0xb9q8qiLsFr0=vr0=vr0dc8meaabaqaciaacaGaaeqabaqabeGadaaakeaadaqiaaqaaiabdsfaujabdcfaqbGaayPadaaaaa@2FC8@ = 316 in which the estimated FDR from the symmetric cutoff method is significantly smaller than that from sam2.20. It can also be seen from Table 5 that the numbers of genes being declared significant positive are much higher than those of genes being declared significant negative for both methods. The results from the bottom row of Table 5 shows that there are no genes with test statistics below -5.5678 (the only significant negative gene has test statistic equal to -5.5678) while there are 22 genes with test statistics larger than 5.5678. This means that the distribution of the test statistics is right skewed, hence resulting in a larger number of induced genes detected as significant.

Table 5 also shows that the asymmetric numbers of induced and repressed called significant by sam2.20 are greatly affected by the asymmetric cutoffs. For example, at TP_
 MathType@MTEF@5@5@+=feaafiart1ev1aaatCvAUfKttLearuWrP9MDH5MBPbIqV92AaeXatLxBI9gBaebbnrfifHhDYfgasaacH8akY=wiFfYdH8Gipec8Eeeu0xXdbba9frFj0=OqFfea0dXdd9vqai=hGuQ8kuc9pgc9s8qqaq=dirpe0xb9q8qiLsFr0=vr0=vr0dc8meaabaqaciaacaGaaeqabaqabeGadaaakeaadaqiaaqaaiabdsfaujabdcfaqbGaayPadaaaaa@2FC8@ = 92, sam2.20 reported 87 significant positive genes and only 5 significant negative genes, compared to 73 and 19 significant positive and negative genes declared by the symmetric cutoff method. It is obvious that the extremely small number (= 5) of significant negative genes from sam2.20 is caused by the use of the lower cutoff (cutlo = -5.1595), which is much larger in its absolute value than the upper cutoff (cutup = 4.0787). We cannot find a reasonable explanation why one should make the declaration of a gene being significant negative much more difficult than being significant positive.

The effect of FP correction (12) can not be observed for both methods in this example. The FDR estimates with FP correction for TP_
 MathType@MTEF@5@5@+=feaafiart1ev1aaatCvAUfKttLearuWrP9MDH5MBPbIqV92AaeXatLxBI9gBaebbnrfifHhDYfgasaacH8akY=wiFfYdH8Gipec8Eeeu0xXdbba9frFj0=OqFfea0dXdd9vqai=hGuQ8kuc9pgc9s8qqaq=dirpe0xb9q8qiLsFr0=vr0=vr0dc8meaabaqaciaacaGaaeqabaqabeGadaaakeaadaqiaaqaaiabdsfaujabdcfaqbGaayPadaaaaa@2FC8@ = 316 are even higher than the original ones. It is not clear why this has happened. Since we do not know which genes are truly DE genes and which genes are truly EE genes for a real data example such as the leukaemia data, it is impossible to know which method indeed works better. However, the paper of Golub et al. [21] provided a list of 15 genes which biologists considered as informative in the classification of leukaemia as ALL or AML. These genes are: *CD11c*, *CD33*, *MB-1*, the leptin receptor, zyxin, Cyclion *D3*, *Op18*, *MCM3*, *Rbap48*, *SNF2*, *TFIIEβ*, *c-Myb*, *E2A*, *HOXA9 *and *TOP2B*. The first three genes encode cell surface proteins for which monoclonal antibodies have been demonstrated to be useful in distinguishing lymphoid from myeloid lineage cells. The leptin receptor provides a new marker of acute leukaemia subtype while the zyxin gene has been shown to encode a LIM domain protein in cell adhesion in fibroblasts. The other genes in the list have been shown to be related to cancer pathogenesis. In Table 5, we reported the numbers of the genes in the list which are included in the genes declared significant by each method at different levels of threshold Δ. It can be seen that the symmetric cutoff method consistently identified more genes from the list than sam2.20 except in the first and last cases.

## 6 Discussion and conclusion

In this paper, we have provided a comprehensive evaluation of SAM, and its R-Package sam2.20. The discrepancies between the algorithms of SAM and sam2.20 are identified. We have also discussed potential drawbacks of SAM and sam2.20. Through comparisons, we have provided a detailed study on the performance of sam2.20 and the symmetric cutoff method and discussed their relative strength. However, it should be pointed out that our comparison was based on the true FDR each method produces given that they identified the same number of significant genes. In practice, the only way of controlling FDR is through its estimated value. Unfortunately, as seen from the simulations, both sam2.20 and the symmetric cutoff method may significantly over-estimate the true FDR. Figures 5, 6 show that the over-estimation problem of the symmetric cutoff method without FP correction is even more severe than sam2.20 under certain situations. This shows the importance of using the proposed FP correction in order to provide efficient control of FDR. It can be seen from the simulations that our proposed FP correction (12) can efficiently correct the over-estimation problem. Nevertheless, there are still some concerns about the use of FP correction (12). One concern is that it may make the under-estimation problem worse if the original method under-estimates the true FDR. Another concern is that the under-estimation problem may become a common problem if the number of genes excluded in the estimation of FDR is too large (namely, if TP_
 MathType@MTEF@5@5@+=feaafiart1ev1aaatCvAUfKttLearuWrP9MDH5MBPbIqV92AaeXatLxBI9gBaebbnrfifHhDYfgasaacH8akY=wiFfYdH8Gipec8Eeeu0xXdbba9frFj0=OqFfea0dXdd9vqai=hGuQ8kuc9pgc9s8qqaq=dirpe0xb9q8qiLsFr0=vr0=vr0dc8meaabaqaciaacaGaaeqabaqabeGadaaakeaadaqiaaqaaiabdsfaujabdcfaqbGaayPadaaaaa@2FC8@ is too large). Such under-estimation would make scientists to report FDR_
 MathType@MTEF@5@5@+=feaafiart1ev1aaatCvAUfKttLearuWrP9MDH5MBPbIqV92AaeXatLxBI9gBaebbnrfifHhDYfgasaacH8akY=wiFfYdH8Gipec8Eeeu0xXdbba9frFj0=OqFfea0dXdd9vqai=hGuQ8kuc9pgc9s8qqaq=dirpe0xb9q8qiLsFr0=vr0=vr0dc8meaabaqaciaacaGaaeqabaqabeGadaaakeaadaqiaaqaaiabdAeagjabdseaejabdkfasbGaayPadaaaaa@30C1@ which is significantly smaller than the true FDR, hence lead to over-estimation of the number of true DE genes among the genes identified as significant by the method. A possible remedy is to find a reasonable estimate of the number of DE genes and then remove those genes in the estimation of FP. Another possible approach is to use the weighted permutation approach or to use the rank scores to reduce the influence of the over-dispersed null scores on the estimation of FDR [15,24]. However, a detailed comparison of these approaches is beyond the scope of this paper. We will investigate the performance of such methods in the future research.

## 7 Methods

### Data sets

The simulated data under Setups 1–3 were generated using **R **[25]. The leukaemia data of Golub et al. were downloaded from the data link provided in [21].

### SAM analysis

SAM analysis was performed according to the algorithm described in [9].

### sam2.20 analysis

sam2.20 analysis was performed using the SAM R-package (Release 2.20) downloaded from the SAM website [26].

### The symmetric cutoff method

The algorithm of the symmetric cutoff method was described in Section 4 of this paper.

## References

[B1] Huber W, Heydebreck A, Vingron M (2003). Analysis of microarray gene expression data. Technical report.

[B2] Dudoit S, Yang HY, Callow JM, Speed PT (2000). Statistical methods for identifying differentially expressed genes in replicated cDNA microarray experiments. Technical report.

[B3] Kerr MK, Martin M, Churchill GA (2000). Analysis of variance for gene expression microarray data. J Comput Biol.

[B4] Thomas JG, Olson JM, Tapscott SJ, Zhao LP (2001). An efficient and robust statistical modelling approach to discover differentially expressed genes using genomic expression profiles. Genome Research.

[B5] Newton MA, Kendziorski CM, Richmond CS, Battner FR, Tsui KW (2001). On differentially variability of expression ratios: improving statistical inference about gene expression changes from microarray data. J Comput Biol.

[B6] Kendziorski CM, Newton M, Lan H, Gould MN (2003). On parametric empirical Bayes methods for comparing multiple groups using replicated gene expression profiles. Stat Med.

[B7] Smyth GK (2004). Linear models and empirical Bayes methods for assessing differential expression in microarray experiments. Statistical Applications in Genetics and Molecular Biology.

[B8] Newton M, Noueiry A, Ahlquist P, Sarkar D (2004). Detecting differential gene expression with a semiparametric hierarchical mixture method. Biostatistics.

[B9] Tusher VG, Tibshirani R, Chu G (2001). Significant analysis of microarrays applied to the ionizing radiation response. Proc Natl Acad Sci USA.

[B10] Efron B, Tibshirani R, Goss V, Chu G (2000). Microarrays and their use in a comparative experiment. Technical Report.

[B11] Efron B, Tibshirani R, Storey JD, Tusher V (2001). Empirical Bayes analysis of a microarray experiment. J Am Stat Assoc.

[B12] Pan W (2003). On the use of permutation in and the performance of a class of nonparametric methods to detect differential gene expression. Bioinformatics.

[B13] Chu G, Narasimhan B, Tibshirani R, Tusher V SAM Significance Analysis of Microarrays-Users guide and technical document. http://www-stat.stanford.edu/~tibs/SAM/sam.pdf.

[B14] Xie Y, Pan W, Khodursky A (2005). A note on using permutation based false discovery rate estimate to compare different analysis methods for microarray data. Bioinformatics.

[B15] Guo X, Pan W (2005). Using weighted permutation scores to detect differential gene expression with microarray data. Journal of Bioinformatics and Computational Biology.

[B16] Delmar P, Robin S, Daudin JJ (2005). VarMixt: efficient variance modelling for the differential analysis of replicated gene expression data. Bioinformatics.

[B17] Zhao Y, Pan W (2002). Modified nonparametric approaches to detecting differentially expressed genes in replicated microarray experiments. Bioinformatics.

[B18] Pan W, Lin J, Le C (2003). A mixture model approach to detecting differentially expressed genes with microarray data. Funct Integr Genomics.

[B19] Larsson O, Wahlestedt C, Timmons AJ (2005). Considerations when using the significance analysis of microarrays (SAM) algorithm. BMC Bioinformatics.

[B20] Zhang S (2006). An improved nonparametric approach for detecting differentially expressed genes with replicated microarray data. Statistical Applications in Genetics and Molecular Biology.

[B21] Golub TR, Slonim DK, Tamayo P, Huard C, Gaasenbeek M, Mesirov JP, Coller H, Loh ML, Downing JR, Caligiuri MA (1999). Molecular classification of cancer: class discovery and class prediction by gene expression monitoring. Science.

[B22] Story JD, Tibshirani R (2003). Statistical significance of genome-wide experiments. Proc Natl Acad Sci USA.

[B23] Rice AJ (1995). Mathematical Statistics and Data Analysis.

[B24] van de Weil MA (2004). Significance Analysis of Microarrays Using Rank Scores. Kwantitatieve Methoden.

[B25] R is a freely available language and environment for statistical computing. http://cran.r-project.org/.

[B26] The SAM R-package is downloaded from the SAM website. http://www-stat.stanford.edu/~tibs/SAM/.

